# Rapid buildup of sympatric species diversity in Alpine whitefish

**DOI:** 10.1002/ece3.4375

**Published:** 2018-08-29

**Authors:** Carmela J. Doenz, David Bittner, Pascal Vonlanthen, Catherine E. Wagner, Ole Seehausen

**Affiliations:** ^1^ Division of Aquatic Ecology Institute of Ecology and Evolution University of Bern Bern Switzerland; ^2^ Department of Fish Ecology & Evolution Center for Ecology, Evolution and Biogeochemistry Eawag Swiss Federal Institute of Aquatic Science and Technology Kastanienbaum Switzerland; ^3^ Fischereiverwaltung Kanton Aargau Aarau Switzerland; ^4^ Aquabios GmbH Les Fermes Cordast Switzerland; ^5^ Biodiversity Institute & Department of Botany University of Wyoming Laramie WY USA

**Keywords:** adaptive radiation, *Coregonus*, evolutionary community assembly, niche partitioning, speciation, stocking

## Abstract

Adaptive radiations in postglacial fish offer excellent settings to study the evolutionary mechanisms involved in the rapid buildup of sympatric species diversity from a single lineage. Here, we address this by exploring the genetic and ecological structure of the largest Alpine whitefish radiation known, that of Lakes Brienz and Thun, using microsatellite data of more than 2000 whitefish caught during extensive species‐targeted and habitat‐randomized fishing campaigns. We find six strongly genetically and ecologically differentiated species, four of which occur in both lakes, and one of which was previously unknown. These four exhibit clines of genetic differentiation that are paralleled in clines of eco‐morphological and reproductive niche differentiation, consistent with models of sympatric ecological speciation along environmental gradients. In Lake Thun, we find two additional species, a profundal specialist and a species introduced in the 1930s from another Alpine whitefish radiation. Strong genetic differentiation between this introduced species and all native species of Lake Thun suggests that reproductive isolation can evolve among allopatric whitefish species within 15,000 years and persist in secondary sympatry. Consistent with speciation theory, we find stronger correlations between genetic and ecological differentiation for sympatrically than for allopatrically evolved species.

## 1 INTRODUCTION

Over the past millennia, glacial dynamics led to dramatic changes in species diversity and ranges in the northern hemisphere (Hewitt, 2004). Glacial dynamics fueled speciation in two ways: ice masses separating populations promoted allopatric divergence, while retreating glaciers uncovering new habitats provided ecological opportunity and also promoted secondary contact. This set the stage for speciation with gene flow and ultimately the coexistence of multiple divergent lineages. Many postglacial fish species emerged from adaptive radiation, a process wherein a suite of species rapidly evolves from a common ancestor through adaptation to different ecological niches (Gavrilets & Losos, 2009). Postglacial fish species pairs (e.g., stickleback, Schluter, 2010; North American whitefish, Bernatchez, 2004) have served as model systems in adaptive radiation research to study its key elements: ecological opportunity, divergent natural selection, resource competition, and ecological speciation, whereby reproductive isolation (RI) evolves as a consequence of divergent natural or ecologically mediated divergent sexual selection (Schluter, 2000). However, the buildup of high sympatric species richness (>2 species) through adaptive radiation, though diagnostic of classic cases of adaptive radiation (Grant & Grant, 2008; Losos, 2009; Seehausen, 2006), has rarely been studied in postglacial radiations.

The buildup of sympatric species diversity through adaptive radiation requires ecological coexistence as well as evolution and/or maintenance of RI, which is often environment‐dependent in evolutionary young adaptive radiations (Schluter, 2000; Seehausen et al., 2008). Several classic examples of adaptive radiation show evidence that environmental factors also limit species richness in radiation‐assembled communities (e.g., Anoles, Rabosky & Glor, 2010; East African cichlids, Wagner, Harmon, & Seehausen, 2014; Hawaiian *Tetragnatha* spiders, Gillespie, 2004). In some radiations, these ecological limits are mainly approached through allopatric speciation and ecological character displacement (Anoles, Losos, 2009; Hawaiian *Tetragnatha* spiders, Gillespie 2005; Darwin's finches, Grant & Grant, 2008), in others through sympatric and parapatric speciation (e.g., East African cichlids, Wagner et al., 2014). Despite occurring in geographic sympatry, speciation in the latter case is still often associated with spatial ecological gradients at a smaller scale (Seehausen et al., 2008). Such fine‐scale structuring of ecological niches has been found to greatly facilitate speciation, both in empirical studies of adaptive radiations in fish (Hudson, Lundsgaard‐Hansen, Lucek, Vonlanthen, & Seehausen, 2016; Seehausen et al., 2008) and in theoretical models (Doebeli & Dieckmann, 2003; Gavrilets, 2004). It also plays a key role for the local coexistence of ecologically differentiated species in adaptive radiations that are assembled through allopatric speciation (Gillespie, 2004; Losos, 2009). However, the mechanisms by which fine‐scale divergence along ecological and reproductive axes contributes to coexistence among many young species within an adaptive radiation remain poorly studied. Likewise, whether this fine‐scale divergence differs between species of sympatric vs. allopatric origin within an adaptive radiation has not been tested. Such differences would be expected, considering that RI is often environment‐dependent in adaptive radiations (Schluter, 2000; Seehausen et al., 2008) and that the evolution of RI is more constrained in sympatry than in allopatry (Kirkpatrick & Ravigné, 2002).

Our study focuses on a postglacial adaptive radiation of Alpine whitefish (*Coregonus* spp.). Due to their independent and parallel diversification in numerous lakes across the northern hemisphere after the last glaciation 10,000–15,000 years ago, whitefish have become an established model system for rapid speciation and adaptive radiation research (Bernatchez, 2004; Bernatchez et al., 2010; Harrod, Mallela, & Kahilainen, 2010; Hudson, Vonlanthen, Müller, & Seehausen, 2007). Sympatric whitefish species have arisen through secondary contact with variable degrees of introgression (North America, Bernatchez & Dodson, 1990), a combination of sympatric and allopatric speciation (Scandinavia, Præbel et al., 2013), and intralacustrine radiations from a hybridogenic ancestral population (Alpine lakes, Hudson, Vonlanthen, & Seehausen, 2010; reviewed in Hudson et al., 2007). Alpine whitefish are monophyletic with respect to their closest relatives outside the Alpine region (Hudson et al., 2010) and have formed more than 30 endemic species over the last 15,000 years in postglacial Alpine lakes (Vonlanthen et al., 2012). This large radiation is structured into multiple smaller monophyletic radiations, each located in a different lake system, hydrologically isolated from all others, and exhibiting similar sets of ecotypes (Hudson et al., 2010). Today, some Alpine lakes harbor up to five sympatric whitefish species (Hudson et al., 2016; Vonlanthen et al., 2012), and whitefish species richness of a lake is positively related to lake‐specific ecological and RI opportunity (Vonlanthen et al., 2012).

Sympatric Alpine whitefish species typically diverge along the water depth gradient and the pelagic–benthic axis. Divergence along the latter is pervasive among sympatric whitefish and generally associated with divergence in benthic–limnetic feeding ecology (Bernatchez, 2004; Kahilainen et al., 2011; Vonlanthen et al., 2012). Gill raker numbers (GRN), a highly heritable trait in whitefish (Bernatchez, 2004), confer important adaptations for feeding at different points along this axis. Low GRN are adaptive for feeding on benthic invertebrates and high GRN for feeding on zooplankton prey, as indicated by trait utility tests with sympatric Alpine whitefish species (Lundsgaard‐Hansen, Matthews, Vonlanthen, Taverna, & Seehausen, 2013; Roesch, Lundsgaard‐Hansen, Vonlanthen, Taverna, & Seehausen, 2013; B. Lundsgaard‐Hansen, C. Rösch, and O. Seehausen, unpublished data) and phenotype–environment correlations (Harrod et al., 2010; Kahilainen et al., 2011). Divergence along water depth occurs both outside the spawning season during feeding (Harrod et al., 2010; Steinmann, 1950), and very strongly during the spawning season on spawning grounds (Hudson et al., 2016; Vonlanthen et al., 2009). Furthermore, temporal (Steinmann, 1950) and spatial spawning segregation across lake habitats (Scandinavia, Østbye, Næsje, Bernatchez, Sandlund, & Hindar, 2005) can contribute to RI in whitefish. In North American whitefish species pairs, genomic incompatibilities that arose during allopatric divergence are likely involved in RI (Dion‐Côté, Symonová, Ráb, & Bernatchez, 2015; Lu & Bernatchez, 1998).

Human influences have strongly shaped today's Alpine whitefish diversity. Around a quarter of the original Swiss whitefish species (8 of 34) have been lost due to eutrophication‐mediated population declines and speciation reversal (Vonlanthen et al., 2012). Additionally, Alpine whitefish have faced fishing pressure for thousands of years (Hüster Plogmann, 2006) and consequently are highly managed by fisheries (Douglas & Brunner, 2002). Since the 1850s, stocking of whitefish has been practiced in Switzerland (Hüster Plogmann, 2006), that is, wild whitefish are caught and killed, sperm and eggs stripped and mixed, fertilized eggs are hatched, and larvae or juveniles are released back into the wild. Stocking within the same lake (“supportive breeding”) is still practiced in many Swiss lakes today, despite its potentially adverse effects on native whitefish through artificial sexual selection and selection imposed by hatchery conditions (Eckmann, 2012). Moreover, these practices also increase opportunities for interspecific hybridization (Hudson et al., 2016). When stocking of whitefish across lakes was finally banned (1946 for Lake Thun, 1991 for all Swiss lakes, BGF 6 I b), translocations of whitefish between Swiss lakes had already occurred (Douglas & Brunner, 2002; Fatio, 1890; Hudson et al., 2010, 2016). These translocations, while problematic from a conservation biology perspective, also provide some excellent opportunities to study evolutionary mechanisms involved in the rapid generation, maintenance, and loss of species diversity in adaptive radiations. Despite this value, this aspect of species translocations has not received much attention from evolutionary biologists.

Here, we study the mechanisms involved in the rapid buildup of sympatric species diversity in Alpine whitefish. Focusing on a large species assemblage in the system of Lakes Thun and Brienz, we combine microsatellite data with ecological and morphological data of more than 2000 whitefish caught in species‐targeted and habitat‐randomized samplings, in order to identify the number of whitefish species in these lakes and their genetic relatedness. Furthermore, we explore the relative importance of different ecological axes (pelagic–benthic dietary axis, nonspawning‐associated depth habitat, spawning depth, and spawning time) for reproductive isolation and coexistence in this large adaptive radiation. Finally, we assess whether the role of these axes for reproductive isolation and coexistence is different for species of presumable sympatric vs. allopatric origin.

## 2 MATERIALS AND METHODS

### 2.1 Study lakes and known whitefish species

Lake Thun (46°40′ N, 7°42′ E; surface area 47.69 km^2^) and Lake Brienz (46°43′ N, 7°58′ E; surface area 29.8 km^2^) are among the deepest (max. depth 214 m and 261 m, respectively) pre‐alpine lakes in Switzerland and among those least affected by anthropogenic eutrophication in the 20th century (Vonlanthen et al., 2012). They arose from the subdivision of a large postglacial lake, “Wendelsee”, a few thousand years ago (Steinmann, 1950). Lakes Brienz and Thun were known to harbor three and five whitefish species, respectively, differing in spawning ecology, GRN, and body size (Supporting Information Table S1; Bittner, 2009; Vonlanthen et al., 2012). All of these species form a monophyletic group, except the species from Lake Thun referred to as “Albock” today, which previous work indicates was introduced from Lake Constance (Douglas, Brunner, & Bernatchez, 1999; Hudson et al., 2010). Ecologically similar species among Lakes Thun and Brienz are each other's closest relatives, suggesting that the radiation predates the separation of the two lakes (Hudson et al., 2010).

### 2.2 Dataset overview

This study includes genetic, ecological, and morphological data for 2,388 individual whitefish collected by habitat stratified random sampling in 2012 or 2014, and by species‐targeted fishing in the years 1950–1975 and 2000–2015 in Lakes Brienz and Thun (this study; Vonlanthen, 2009; Bittner, 2009; Vonlanthen et al., 2012; Supporting Information Table S2). Stratified random fishing was conducted under a research program for the assessment of fish diversity in pre‐alpine lakes and involved quantitative and taxonomically unbiased sampling across the entire lake (Alexander et al., 2015; Vonlanthen & Périat, 2013; Vonlanthen et al., 2015). Contemporary species‐targeted fishing was conducted by commercial local fishermen during spawning seasons on spawning grounds known to local fishermen and targeted reproductively active fish of known whitefish species (Supporting Information Table S1). Historical fishing targeted known species and was conducted from 1952 to 1972, corresponding to the phase of increasing phosphorus concentrations in these lakes (Vonlanthen et al., 2012).

### 2.3 DNA extraction, microsatellite amplification, and genotyping

In this study, we genotyped 1,050 individual whitefish at 10 microsatellite loci (CoCl49, CoCl68, CoCl6, C2‐157, CoCl61, CoCl45 and BWF‐2, CoCl4, CoCl18, CoCl10 (Turgeon, Estoup, & Bernatchez, 1999; Rogers, Marchand, & Bernatchez, 2004; Patton, Gallaway, Fechhelm, & Cronin, 1997)) that are located on several different whitefish linkage groups (Rogers, Isabel, & Bernatchez, 2007), and combined these data with genotypes of 1,338 whitefish from previous studies (Vonlanthen, 2009; Bittner, 2009; Vonlanthen et al., 2012; Supporting Information Table S2). Detailed information about DNA extraction and microsatellite amplification for this study is provided in Supporting Information Table S2.

We also newly extracted and re‐genotyped 122 fish from previous studies to verify that genotyping was consistent across different scorers and sequencing machines, and found >95% genotyping agreement (Supporting Information Appendix S1). For our final dataset, we excluded all individuals that had missing genotypes at more than 4 loci.

### 2.4 Identification of genetic clusters

Because the study by Hudson et al. (2010) suggested that whitefish from Lakes Thun and Brienz diversified in the larger postglacial lake “Wendelsee” that comprised both modern lakes prior to their separation a few thousand years ago, we sought to identify the number of distinct genetic clusters of whitefish by combining all contemporary and historical samples from both lakes (*n* = 2388).

To find the most likely number of genetic clusters (K), we conducted a hierarchical cluster analysis (Coulon et al., 2008) using the individual‐based Bayesian clustering algorithm implemented in STRUCTURE (Pritchard, Stephens, & Donnelly, 2000). In brief, we determined the most likely *K* for the full dataset, then the most likely *K* within each of the data subsets suggested by the previous analysis, and so forth until all subsets supported a value of *K* = 1 (see Supporting Information Figure S1 for details). If LnP(D) of *K* = 2 and *K* = 1 were very similar in this analysis, we tested the plausibility of *K* = 2 by exploring the relationship between genetic assignments and morphological (GRN) or ecological (spawning depth) data (see Supporting Information Figures S1 and S2 for details). To determine correspondence of genetic clusters to known species, we assessed how individuals from targeted samplings of known species were distributed among them.

To obtain genetic assignment proportions for individuals, we used representative individuals from each of the six clusters identified before as reference populations and assigned all remaining individuals to these references with the “USEPOPINFO” and “POPFLAG” model in STRUCTURE (for details see Supporting Information Figure S1).

### 2.5 Genetic structure of the whitefish community

We visualized genotypic variation in contemporary whitefish communities in three ways. First, we performed a genetic PCA based on individual allele frequencies including all whitefish from both lakes (*N* = 2388) using the “dudi.pca” function of the R package “adegenet” (Jombart, 2008) with default settings (centering and scaling the data). Missing data were replaced by mean allele frequencies. Second, we displayed STRUCTURE assignments from the analysis using reference populations in a tetrahedron using the “plot3D.acomp” function in the R package “compositions” (Van den Boogaart, Tolosana, & Bren, 2014). We restricted these plots to the four clusters found in both lakes and hence for Lake Thun only included individuals whose sum of assignment likelihoods was >0.85 for those four clusters together. Finally, we plotted frequency distributions of STRUCTURE assignments for all possible pairs of genetic clusters within Lakes Thun and Brienz. We included individuals whose sum of assignment likelihoods to the two clusters under consideration together was >0.8.

### 2.6 Genetic, morphological, and ecological differences between species

To estimate neutral genetic differentiation among the clusters inferred in the STRUCTURE assignment, we calculated multilocus pairwise *F*_ST_ values over 1,000 permutations in ARLEQUIN v.3.11 (Excoffier, Laval, & Schneider, 2005) for each lake. We calculated multilocus *F*_ST_s using (i) all contemporary individuals grouped by their highest genetic assignment proportion, or using (ii) only contemporary individuals with high assignment proportion (>0.7) to one genetic cluster. For these two sets of groups, we also calculated the mean number of private alleles per locus in ADZE‐1.0 (Szpiech, Jakobsson, & Rosenberg, 2008). We further calculated pairwise *F*_ST_ for all loci separately in ARLEQUIN for the first set of groups to assess patterns in locus‐specific differentiation. We assessed whether the proportion of unclear assignments (highest assignment proportion <0.7) differed between lakes using the fish from stratified random fishing.

To test whether genetic clusters differed along major ecological and reproductive axes of whitefish divergence (diet‐related GRN, nonspawning‐associated depth habitat (estimated from capture depth in the benthic or the pelagic lake zone in autumn), spawning depth and spawning time), we performed Kruskal–Wallis and post hoc Dunn's test with Holm's method to account for multiple testing. We conducted these analyses separately per lake using only clearly assigned individuals (highest assignment proportion >0.7) from contemporary samplings to avoid potential misassignment or hybrids. All analyses, if not stated differently, were performed in R 3.2.1 (R Core Team, 2015).

To test whether geographic structure contributed to between‐species differentiation, we assessed for each lake the correlation between individual differences in cluster membership (0 same cluster, 1 different cluster) and geographic distance of spawning location in Mantel tests and partial Mantel tests correcting for spawning depth or spawning time using the R package “ecodist” (Goslee & Urban, 2007). To correct for both spawning time and spawning depth, we took residuals of Mantel tests between cluster membership and spawning depth using the function “multi.mantel” of the R package “phytools” (Revell, 2012), and used those residuals in partial Mantel tests with geography while correcting for spawning time. For Lake Thun, we performed these tests for all contemporary individuals (*n* = 918) and only for species shared with Lake Brienz (including only individuals whose sum of assignment likelihood for these four species was >0.85, *n* = 356).

### 2.7 Intraspecific variation within and between lakes

To explore intraspecific structure within lakes, we performed Mantel tests between individual genetic and ecological data for each genetic cluster within each lake (for details see Supporting Information Appendix S2). To explore intraspecific structure between lakes, we calculated *F*_ST_ in Arlequin and compared phenotypic and ecological data in Wilcoxon tests using clearly assigned individuals from contemporary samplings.

### 2.8 Origins of an introduced species

We explored the origin of the whitefish species today named “Albock” in Lake Thun in more detail because of previous evidence that it was introduced from Lake Constance (Douglas et al., 1999; Hudson et al., 2010). We therefore calculated multilocus pairwise *F*_ST_ between “Albock” from Lake Thun (reference population) and four species from Lake Constance's pre‐eutrophication/eutrophication period sampled in 1929–1973 (Vonlanthen et al., 2012). We used STRUCTURE (*K* = 4, 5 replicates, 100,000 burn‐in, 100,000 MCMC steps) to assign individuals from Lake Constance to species according to their maximum assignment likelihood. Because these historical samples contained many missing data for markers Cocl10 and Cocl61, we used only the remaining eight markers. Furthermore, we searched for historical records of whitefish stocking in the archives of the fisheries association (Oberländischer Fischereiverein, Interlaken) that was responsible for fish propagation in Lakes Thun/Brienz before stocking of allochthonous whitefish was banned in 1946.

### 2.9 Testing the roles of major ecological axes for whitefish coexistence and RI

To test the importance of different axes of whitefish divergence for coexistence and RI, we compared *P*_ST_s (a phenotypic analog of *F*_ST_) of capture depth in autumn (outside spawning seasons) to *P*_ST_s of GRN, and *P*_ST_s of spawning time to *P*_ST_s of spawning depth, in paired *t*‐tests for each lake. *P*_ST_ was calculated following the method of Kaeuffer, Peichel, Bolnick, and Hendry (2012) using 1000 resampling permutations. We used all individuals of contemporary samplings and assigned individuals to species based on their major assignment likelihood (*N* = 2215).

Furthermore, if divergence along these axes was important for maintaining RI, we predicted a positive association between the degree of ecological and neutral genetic differentiation. To test this, we performed Mantel tests between *F*_ST_s and *P*_ST_s for each ecological axis within each lake. We also explored whether combining important ecological axes predicted *F*_ST_ better than any single axis alone using partial Mantel tests implemented in the R package “ecodist”.

Focusing on the four species shared between lakes, we performed Mantel tests between multilocus individual genetic distance (Rousset, 2000; calculated in SPAGeDI v. 1.5a Hardy & Vekemans, 2002) and individual differences in spawning depth, spawning time, geographic distance of spawning locations, or GRN. We also performed partial Mantel tests by correcting for one of these factors. Additionally, for these same four species, we tested whether genetic variation was structured into distinct entities, instead of being continuously distributed along an environmental gradient. We therefore performed partial Mantel tests between individual genetic distances and individual differences in cluster membership while correcting for spawning depth, geography, GRN, and spawning time within each lake (see Supporting Information Appendix S3 for details).

## 3 RESULTS

### 3.1 Six sympatric whitefish species

Using hierarchical STRUCTURE analysis, we found two genetic clusters of whitefish only in Lake Thun, which corresponded to the known species *C. alpinus* Fatio 1885 and *C*. sp. “Albock”. Two further clusters were found in both lakes (Supporting Information Figures S1, S2). Nonspatial subdivision of these two clusters into two groups each (outlined below) yielded four groups (*C*. sp. “Balchen1”, *C*. sp. “Balchen2”, *C*. sp. “Felchen”, *C. albellus* Fatio 1890), one of which was previously unknown (*C*. sp. “Balchen2”). These four groups were found in both lakes, resulting in four sympatric genetic groups in Lake Brienz and six in Lake Thun. Because all six genetic groups were ecologically and genetically clearly distinct in full sympatry, we refer to them from now on as biological species and use their scientific names for the described species *C. alpinus*, and *C. albellus* and cheironyms for the undescribed: *C*. sp. “Albock”, *C*. sp. “Balchen1”, *C*. sp. “Balchen2”, *C*. sp. “Felchen.”

In the “Balchen” cluster, STRUCTURE analyses for *K* = 2 revealed a continuous distribution of genetic assignments which were correlated with GRN in both lakes (*p* < 0.001, Supporting Information Figure S3a,b). The two genetic groups differed significantly in GRN and spawning depth within each lake, and more so in Lake Brienz, where GRN has a bimodal distribution. We refer to the genetic “Balchen” group with low GRN and shallow spawning depth as *C*. sp. “Balchen1”, and to the other as *C*. sp. “Balchen2”.

In the *C*. sp. “Felchen”/*C. albellus* cluster, STRUCTURE analyses for *K* = 2 also revealed a continuous distribution of genetic assignments in both lakes that were correlated with GRN in Lake Brienz (*p* < 0.001), but not in Lake Thun (*p* = 0.137) (Supporting Information Figure S3c,d). These two genetic groups differed significantly in GRN in each lake (*p* < 0.01), and in spawning depth in Lake Brienz (*p* = 0.006), but not in Lake Thun (*p* = 0.326) (Supporting Information Figure S3c,d). Following previous work (Steinmann, 1950; Supporting Information Table S1), we refer to the species with higher GRN and greater spawning depth as *C. albellus*, and to the other as *C*. sp. “Felchen”. In support of these groups, when STRUCTURE analyses with similar sample sizes of *C*. sp. “Felchen” and *C. albellus* from known spawning sites of these species in Lake Brienz were performed, *K* = 2 was most likely and genetic assignments matched with field identifications (Supporting Information Figure S4).

### 3.2 Evidence for genetic differentiation and RI among all sympatric species

All species were strongly genetically differentiated from each other with pairwise *F*_ST_s of 0.12–0.39 and 0.05–0.34 when considering clearly assigned or all contemporary individuals, respectively (Table 1, Supporting Information Table S3). In both lakes, *F*_ST_s were highest between *C. albellus* and *C*. sp. “Balchen1” and lowest between *C. albellus* and *C*. sp. “Felchen”, when all individuals were included. Locus by locus *F*_ST_s revealed that in all pairwise comparisons, the majority of loci contributed to genetic differentiation (six to ten loci with significant *F*_ST_, Supporting Information Tables S4, S5) indicating genome‐wide differentiation, and therefore little or no current gene flow among sympatric species.

**Table 1:** Genetic differentiation (*F*_ST_) between contemporary genetic groups (individuals are assigned based on their maximum assignment proportion) within Lake Thun (below diagonal) and within Lake Brienz (above diagonal). All *F*_ST_s are highly significant (*p* < 0.001). Sample sizes are given in brackets, left for Lake Thun, in the top row for Lake Brienz

Genetic group	*C*. sp. “Balchen1” (50)	*C*. sp. “Balchen2” (60)	*C*. sp. “Felchen” (164)	*C. albellus* (469)	*C. alpinus*
*C*. sp. “Balchen1” (142)	–	0.11	0.22	0.34	–
*C*. sp. “Balchen2” (158)	0.08	–	0.09	0.21	–
*C*. sp. “Felchen” (200)	0.19	0.07	–	0.06	–
*C. albellus* (383)	0.27	0.14	0.05	–	–
*C. alpinus* (275)	0.2	0.13	0.18	0.2	–
*C*. sp. “Albock” (307)	0.12	0.09	0.16	0.21	0.08

Focusing on the four species shared between lakes, pairwise *F*_ST_s were consistently lower in Lake Thun than in Lake Brienz when considering all contemporary individuals (Table 1). Concomitantly, the proportion of individuals with low maximum genetic assignment likelihood was higher in Lake Thun, both within species and overall (Supporting Information Table S6).

Private alleles were present in all species, independent of whether species were analyzed within or combined across lakes (Supporting Information Figure S5). The mean number of private alleles per locus ranged from 0.18 (*C*. sp. “Balchen1”) to 0.50 (*C*. sp. “Balchen2”) in Lake Brienz, and from 0.04 (*C*. sp. “Balchen1”) to 0.64 (*C*. sp. “Albock”) in Lake Thun, when considering all individuals. This pattern remained when considering only clearly assigned individuals and when combining individuals from both lakes (Supporting Information Figure S5).

We found bimodal genetic assignment distributions for all pairwise species comparisons (Figure 2), indicating RI among all species (Jiggins & Mallet, 2000). The frequency of intermediate assignment likelihoods varied between species pairs. In both lakes, individuals with intermediate assignment were frequently found between *C. albellus*–*C*. sp. “Felchen”, *C*. sp. “Felchen”–*C*. sp. “Balchen2”, and *C*. sp. “Balchen2”–*C*. sp. “Balchen1”. Intermediate assignments between *C*. sp. *“*Albock” and either “Balchen” species were rather common, while they were rare between *C*. sp. “Albock” and *C. albellus* or *C*. sp. “Felchen”.

In both lakes, data from the 1950 to 1970 showed similar distributions of assignment likelihoods as the contemporary data but sample sizes were low (Supporting Information Figure S6).

### 3.3 Reproductive and ecological niche differentiation among sympatric species

**Figure 1:**
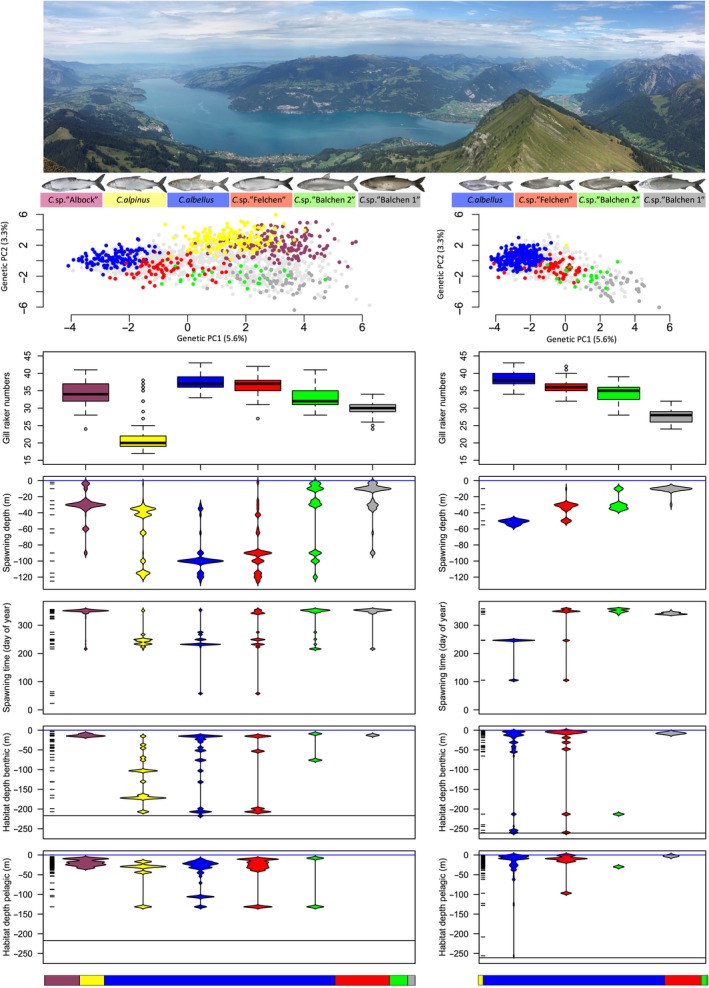
Genetic, morphological, and ecological differences among whitefish species from Lake Thun (left) and Brienz (right). In the genetic PCA, only individuals of clear species assignment in the Structure assignment analysis are colored. Lake bottom is indicated by horizontal black line, lake surface by horizontal blue line. The distribution of where and when nets were set is indicated along the *y*‐axis. Relative species abundances, corrected for habitat volume according to the method of Alexander et al. (2015) are shown at the bottom

In both lakes, species significantly differed in spawning depth, spawning time and GRN (Figure 1, Supporting Information Tables S7‐S9). In Lake Brienz, all but one pairwise comparison of spawning depth were significant (Supporting Information Table S5). *C. albellus* spawned at the greatest depth, followed by *C*. sp. “Felchen” and *C*. sp. “Balchen2”, while *C*. sp. “Balchen1” spawned the shallowest (Figure 1). In Lake Thun, most pairwise comparisons were significant (for details see Supporting Information Table S7). *C. albellus* and *C*. sp. “Felchen” spawned at the greatest depths, *C. alpinus* at intermediate and great depth, *C*. sp. “Albock” at intermediate depths, *C*. sp. “Balchen2” at a range of depths, and *C*. sp. “Balchen1” at very shallow depths (Figure 1).

For spawning time, all but one pairwise species comparison were significant in Lake Brienz (Supporting Information Table S8), and most of them were significant in Lake Thun (for details see Supporting Information Table S8). *C*. sp. “Balchen1”, *C*. sp. “Balchen2” and *C*. sp. “Albock” spawned in winter, *C. albellus* and *C. alpinus* in autumn, while *C*. sp. “Felchen” spawned in winter in Lake Brienz, but in autumn in Lake Thun (Figure 1).

In Lake Brienz, *C. albellus* had the highest GRN, followed by *C*. sp. “Felchen”, *C*. sp. “Balchen2” and *C*. sp. “Balchen1” (Figure 1). All but one pairwise comparison were highly significant (Supporting Information Table S9). In Lake Thun, *C. alpinus* had substantially lower GRN than any other species, while *C*. sp. “Albock” was intermediate in GRN to *C*. sp. “Felchen” and *C*. sp. “Balchen2” (Figure 1). All except two pairwise comparisons were significant (*p* < 0.05, Supporting Information Table S9).

Species did not significantly differ in mean capture depth in autumn (when most species except *C. albellus* and *C. alpinus* were not spawning), neither in the benthic, nor in the pelagic zone (Supporting Information Tables S10, S11). Nevertheless, sympatric species tended to differ in their depth ranges (Supporting Information Figure S7, Appendix S4).

In both lakes, geographic distance alone explained very small amounts of species differences (Supporting Information Table S12). Focusing on the four species shared between lakes, in both lakes geographic distance did not explain species differences when accounting for spawning depth differences. Considering all species of Lake Thun, geography explained only a very small proportion of between‐species differences when accounting for spawning depth and spawning time differences (Supporting Information Table S12).

*C. albellus* and *C*. sp. “Felchen” showed weak intraspecific genetic differentiation between spawning grounds in Lake Thun, but these patterns lost significance after correction for multiple testing (Supporting Information Table S13). Between lakes, conspecific populations did not differ genetically, and only very few phenotypic and ecological differences existed (Figure 1, Supporting Information Table S14, Appendix S5).

### 3.4 An introduced pelagic species from another radiation

Comparing *C*. sp. “Albock” of Lake Thun with the four species from Lake Constance, we found that it was significantly genetically differentiated from all four, but was least strongly differentiated from *C. macrophthalmus* Nüsslin 1882 (“Gangfisch”) (*F*_ST_ = 0.028, *p* < 0.0001, Supporting Information Table S15). Historical documents confirm that whitefish from Lake Constance were massively stocked into Lake Thun: hatcheries at Lake Thun obtained 1 million fertilized whitefish eggs from Lake Constance in December 1934, of which 756,000 whitefish larvae were hatched and introduced into Lake Thun in 1935 (Supporting Information Figure S15). According to these reports, these eggs derived from “Blaufelchen”, that is, *C. wartmanni* Bloch 1784. Our data and the historical records hence do not agree on the identity of the species introduced from Lake Constance. However, in either case, the introduced species was a pelagic species from Lake Constance (see Supporting Information Appendix S6 for additional details).

### 3.5 Spawning depth and gill raker numbers predict genetic differentiation

Ecological differentiation (*P*_ST_) between species was stronger along the benthic–pelagic, diet‐related resource axis (GRN) than along habitat depth outside spawning (autumn data) in both lakes (both *p* < 0.02, Supporting Information Figure S8a). *P*_ST_s of habitat depth outside spawning were frequently not significantly different from zero for native species of Lakes Brienz (5 of 6 comparisons) and Thun (5 of 10 comparisons), but they were mostly significant for pairwise tests between the introduced species and the native species from Lake Thun (4 of 5 comparisons) (Supporting Information Table S16). *P*_ST_s of spawning depth were significantly greater than *P*_ST_s of spawning time in Lake Brienz (*p* = 0.027). The same was true in Lake Thun for the four species that occur in both lakes (*p* = 0.047), but not when considering all species (*p* = 0.162) (Supporting Information Figure S8b).

In both lakes, Mantel tests revealed that *F*_ST_s were significantly positively related to *P*_ST_s of spawning depth and GRN, but not to *P*_ST_s of habitat depth in autumn (Figure 3a,b,c). In Lake Thun, but not in Lake Brienz, *F*_ST_ tended to be related to *P*_ST_ of spawning time (Figure 3c). In Lake Thun, *P*_ST_ of GRN explained significant residual variation in *F*_ST_ when correcting for *P*_ST_ of spawning depth, while this association was barely significant in Lake Brienz (Figure 3e). In both lakes, *P*_ST_ of spawning time did not explain residual variation in *F*_ST_ when correcting for *P*_ST_ of spawning depth or GRN (partial Mantel test correcting for spawning depth Thun: *r* = −0.04, *p* = 0.92; Brienz *r* = −0.43, *p* = 0.49; or GRN Thun *r* = 0.42, *p* = 0.14; Brienz *r* = 0.45, *p* = 0.40). In Lake Thun, associations between *F*_ST_ and *P*_ST_ of spawning depth, time and GRN became stronger when considering only the four species that occur in both lakes and have speciated in situ in Lake “Wendelsee” (Figure 3).

Focusing on the four species that occur in both lakes, Mantel and partial Mantel tests at the individual level revealed that spawning depth, spawning time and GRN explained considerable genetic variation in both lakes, while geographic location of spawning sites was of very minor importance (Supporting Information Table S17). In Lake Brienz, spawning depth was of primary importance, while in Lake Thun, spawning depth, time, and GRN were similarly important.

Considering these same four species, individual differences in genetic cluster (species) membership explained considerable and significant residual variation in individual genetic distances after differences in spawning depth, geographic location of spawning sites, GRN, spawning time, or all four factors together were taken into account (Supporting Information Table S18).

## 4 DISCUSSION

In Lakes Thun and Brienz, we find evidence for the most speciose known Alpine whitefish assemblage consisting of six fully sympatric whitefish species. Among the four species occurring in both lakes, we find a strong association between genetic and adaptive phenotypic species differentiation structured along water depth of spawning sites. Given the previously discovered monophyly of this species group (Hudson et al., 2010), this is consistent with theoretical models of sympatric speciation along environmental gradients (Doebeli & Dieckmann, 2003). In Lake Thun, we additionally find a native profundal specialist species and whitefish species introduced in the 1930s from another lake radiation. We find evidence for the maintenance of RI in sympatry between this introduced species and all native species, demonstrating a clear, but rarely reported case of allopatric speciation in Alpine whitefish. Consistent with speciation theory, niche differentiation and RI are more strongly correlated among species of sympatric than among those of allopatric origin. We discuss each of these findings below.

### 4.1 The shape of a speciose Alpine whitefish radiation

We found evidence for six ecologically and genetically differentiated sympatric species of whitefish in the lake system of Thun and Brienz: *C*. sp. “Balchen1”, *C*. sp. “Balchen2”, *C*. sp. “Felchen”, *C. albellus, C. alpinus, C*. sp. “Albock”. The first five species are native, the second of which was previously unknown (see Supporting Information Appendix S7 for taxonomic considerations), while the sixth was introduced from the radiation of Lake Constance.

Clear evidence for RI among all of them (as indicated by neutral genetic differentiation across multiple loci and private alleles) together with morphological differences and strong ecological and reproductive niche differentiation in full sympatry provide evidence for them being real biological species, and not locally adapted populations of one species. Three of these species were taxonomically described long ago (Fatio, 1885, 1890; Kottelat, 1997). We point out that the presence of multiple species of whitefish in these lakes has been known for centuries (e.g., Fischerordnungen from 1673 in Rennefahrt, 1967; Helvetischer Almanach, Zürich, 1819), and today's local fishermen are well aware of this diversity. Hence, we emphasize that the diversity we here describe is by no means cryptic.

Note that genetic structure of Alpine whitefish in Lakes Thun and Brienz cannot be attributed to spatial population structure, since geography, contrary to ecological variables, explained very little genetic structure within species (Supporting Information Table S13) and between all species in Lake Thun (Supporting Information Table S12). Within both lakes, none of the between‐species genetic structure was explained by geography, when considering the four species occurring in both lakes (Supporting Information Table S12). This is consistent with earlier results from other Alpine whitefish radiations (Hudson et al., 2016; Vonlanthen et al., 2009). This may indicate that for highly mobile fish such as these, these postglacial lakes are too small for geographical genetic structure to arise.

With six fully sympatric whitefish species, Lake Thun harbors the highest number of known Alpine whitefish species (Lake Lucerne has five fully sympatric species; Hudson et al., 2016). The only lake with higher whitefish species richness is Lake Onega in Russia with nine known species (Kottelat & Freyhof, 2007), whose species status and degree of sympatry is however unclear. Note that the six species we identify here are potentially a minimum estimate for the actual whitefish species richness of Lakes Thun/Brienz, because the modest number of neutral microsatellite loci restricts us to detect common and clearly differentiated species (Supporting Information Appendix S8). Due to the same reason, our capacity to confidently assign individuals to young species that still have the potential to hybridize is also limited. Genomic data show similar levels of species differentiation to those we observe with ten microsatellite markers (P.G.D. Feulner and O. Seehausen, unpublished data) and will help to shed more light on species boundaries in these large whitefish radiations.

Lake Thun attained its high sympatric whitefish species richness through two distinct assembly mechanisms: (i) *in situ* evolution, and (ii) addition of species that are of allopatric in origin. We discuss each of these mechanisms below. First, the four species found in both Lakes Thun and Brienz are genetically and eco‐phenotypically very similar between lakes, as expected for species originating in the larger postglacial lake “Wendelsee” that comprised both modern lakes. These four species likely arose *in situ* (Hudson et al., 2010) and can be arranged along a discontinuous cline of neutral genetic differentiation that is paralleled by clines in ecological (gill raker numbers) and reproductive niches (spawning depth and spawning time) (Figure 1). A remarkably similar species structure by spawning depth is found for three (potentially four when including *C. nobilis*) whitefish species of Lake Lucerne (Hudson et al., 2016) and the two of Lake Neuchâtel (Vonlanthen et al., 2009). The main difference among these radiations is the stronger genetic differentiation in Lakes Thun and Brienz (max. F_ST_ Neuchâtel 0.07, Lucerne 0.12, Thun 0.27, Brienz 0.34), which may be attributable to the great depth of these lakes providing extensive reproductive and ecological niche space. Furthermore, Lakes Thun and Brienz were less affected by eutrophication than Lakes Neuchâtel and Lucerne (Vonlanthen et al., 2012), which allowed for continuous accessibility of the full depth range, and therefore facilitated both temporal and spatial spawning segregation (Figure 1). Although we found spawning depth to be more important for RI than spawning time, the parallelism of these two dimensions likely strengthens RI among species (Supporting Information Appendix S9). Notably, even very mild eutrophication seems to have increased gene flow among species in Lake Thun compared to the less affected Lake Brienz (Supporting Information Appendix S10; Bittner, Excoffier, & Largiadèr, 2010).

We point out that isolation by spawning depth alone is unlikely to explain the genetic structure among the four whitefish species shared between Lakes Brienz and Thun. Simple isolation by spawning depth would result in a continuous distribution of genotypes along depth, and clustering programs like Structure are known to sometimes infer multiple clusters from continuous data (Frantz, Cellina, Krier, Schley, & Burke, 2009). However, cluster membership explained considerable residual genetic variation among individuals of the four species after we had taken differences in spawning depth, geography, GRN, and spawning time into account (Supporting Information Table S18). This is evidence that the whitefish of Lakes Thun and Brienz are not a genetic continuum, but genetically distinct species arranged along a depth gradient during spawning, but with considerable overlap.

Lake Thun also harbors two whitefish species not shared with Lake Brienz that are likely both of allopatric origin and do not fall along the eco‐phenotypic cline of the four other species. One is *C*. sp. “Albock”, a species that has been introduced from Lake Constance, and has remained strongly genetically differentiated from all native whitefish species in Lake Thun. An extensive introduction of Lake Constance whitefish into Lake Thun in the 1930s is well documented, suggesting that *C*. sp. “Albock” likely derives from this event. This introduced species was below detection limit in our samples from 1958 to 1972 and is now so abundant in Lake Thun that it has also become commercially important (Supporting Information Appendix S11).

**Figure 2:**
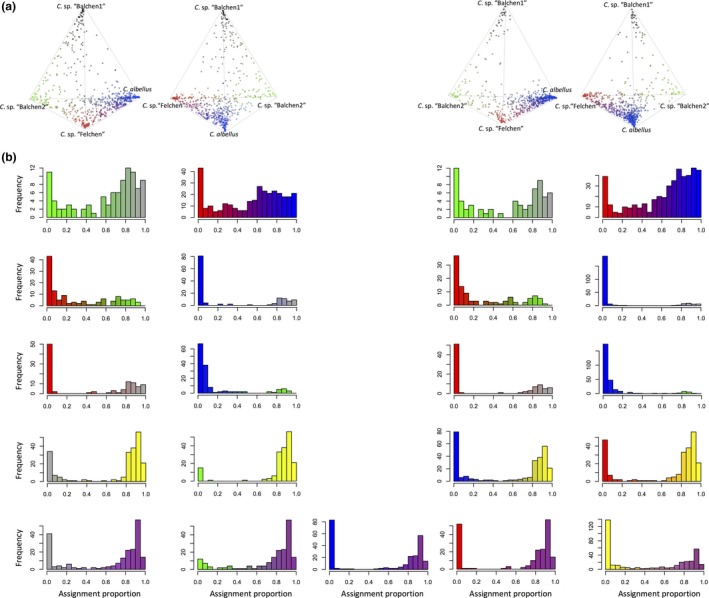
Genetic structure of today's whitefish community in Lake Thun (left) and Brienz (right). Colors correspond to species as indicated in Figure 1. (a) Tetrahedral plots showing the genotypic distribution of the contemporary whitefish communities. For each lake, the same tetrahedral plot is displayed from different angles. The location of an individual is determined by its STRUCTURE assignment proportions obtained from the assignment analysis. Corners correspond to 100% assignment to a cluster, and color reflects the combination of assignment proportions for the different clusters. (b) Frequency distributions of STRUCTURE assignments for all possible species pairs from Lake Thun (left and two bottom panels) and Brienz (right). We only used individuals whose sum of assignment likelihood to the two genetic clusters under consideration was >0.8. The frequency distribution of assignment proportions was plotted for one of the clusters under consideration (at position 1)

**Figure 3:**
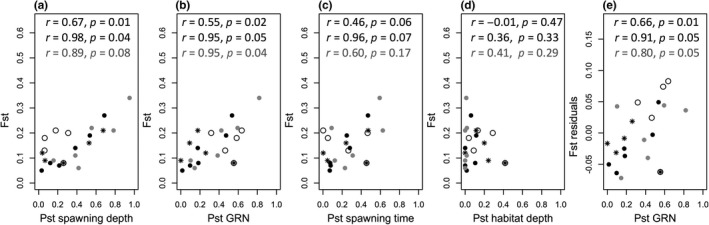
Correlations between FST and PST of (a) spawning depth, (b) gill raker numbers (GRN), (c) spawning time, (d) habitat depth in autumn, and (e) GRN when correcting FST for spawning depth in Lake Thun (black) or Brienz (gray). Individual points are pairwise comparisons between sympatric species, results of Mantel tests (a–d) or partial Mantel tests (e) are indicated on top. For Lake Thun, the first result includes all species, the second excludes the two of potential allopatric origin, *C*. sp. “Albock” and *C. alpinus*. Comparisons with the former species are indicated with stars, those with the latter with open circles

The other is the native profundal *C. alpinus*, which is the only remaining Alpine whitefish species of its ecomorph in all of Switzerland, as its ecological equivalent in Lake Constance, *C. gutturosus* Gmelin 1818, went extinct during eutrophication (Vonlanthen et al., 2012). Note that it is unlikely that *C. alpinus* derives from a recent human‐mediated introduction of *C. gutturosus* from Lake Constance, because these two species are genetically clearly distinct (*F*_ST_ = 0.174), exhibit different microstructure of gill rakers (Steinmann, 1950) and *C. alpinus* was documented to occur in Lake Thun before major stocking with whitefish from Lake Constance occurred (Bureausitzung Oberländischer Fischereiverein 1932). Several lines of evidence are consistent with *C. alpinus* being potentially of allopatric origin: First, a phylogenetic tree based on genomic markers provided low support for grouping *C. alpinus* with the other species from Lakes Thun and Brienz (Hudson et al., 2010). Second, the distributions of genetic assignment proportions of *C. alpinus* are similar to those of the introduced *C*. sp. “Albock”, showing fewer unclearly assigned individuals than the four other species (Figure 2b). Finally, both *C. alpinus* and the introduced species showed only a weak relationship between genetic and ecological differentiation against the other species (Figure 3), suggesting a different speciation mechanism.

### 4.2 Sympatric speciation along an environmental gradient

Given the monophyly of the whitefish radiation shared between Lakes Thun and Brienz (Hudson et al., 2010), the strong correlation between genetic and adaptive phenotypic species differentiation structured along spawning depth among the four species occurring in both lakes is consistent with sympatric ecological speciation along a spawning depth gradient, the mode of speciation proposed in previous studies on Alpine whitefish radiations (Hudson et al., 2016; Ingram, Hudson, Vonlanthen, & Seehausen, 2012; Vonlanthen et al., 2009). In theoretical models of sympatric/parapatric speciation along environmental gradients, dispersal limitation along the gradient and assortative mating are crucial for speciation to occur (Doebeli & Dieckmann, 2003; Kawata, Shoji, Kawamura, & Seehausen, 2007). As a previous study pointed out (Hudson et al., 2016), despite closely matching the patterns predicted by these models, Alpine whitefish do not obviously fulfill the model assumption of dispersal limitation along lake depth. We here find clear evidence for the absence of such dispersal limitation, since most whitefish species were distributed across the entire lake depth and largely overlapped in depth range during the stratified random fishing (e.g., outside the spawning season, Figure 3). Furthermore, Alpine whitefish also differ from other young fish species pairs of proposed sympatric origin that show segregation along water depth (e.g., haplochromine cichlids in Lake Massoko (Malinsky et al., 2015) or Lake Victoria (Seehausen et al., 2008)). In cichlids, individuals live, feed and breed year‐round at the same depth, but this does not seem to be the case for Alpine whitefish, which show species segregation along water depth only during spawning time (Figure 3). Hence, the spawning depth gradient and the phenotypic, resource‐related benthic–pelagic gradient in Alpine whitefish must be linked differently than through dispersal limitation or divergent selection along depth.

Alpine whitefish likely directly fulfill the second key element of theoretical models of speciation along environmental gradients, which is nonrandom mating. If these models assume random mating, a continuous phenotypic (and genetic) cline evolves along the environmental gradient (Endler, 1977; Barton, 1999; Doebeli & Dieckmann, 2003). In the whitefish, we do not find a genotypic cline (Table S18), and thus, we infer that assortative mating likely occurs among whitefish species on the spawning depth gradients of Lakes Thun and Brienz. Consistent with this, assortative mating between benthic and limnetic whitefish species from Lake Lucerne has been observed in large outdoor experimental ponds (B. Lundsgaard‐Hansen, C. Rösch, & O. Seehausen, unpublished data).

### 4.3 Postglacial allopatric speciation and maintenance of RI in sympatry

The occurrence of a whitefish species from the Lake Constance radiation as a clearly distinct genetic group (Figure 2) in Lake Thun at least 20 generations after its introduction implies strong RI from all native whitefish species of Lake Thun. No recent allochthonous introductions are documented, and furthermore, they are forbidden by the local fisheries authorities since 1946 (Douglas et al., 1999) and by federal law since 1991 (BGF 6 I b). This and the stable coexistence of multiple introduced whitefish species with allopatric histories in lakes of the Southern Alps (Hudson, Vonlanthen, Lundsgaard‐Hansen, Denis, & Seehausen, 2008) suggest that strong RI that is independent of the local environment in which it evolved, can evolve among allopatric whitefish species in less than 15,000 years. Allopatric speciation and ecological character displacement upon secondary contact is the dominant speciation mechanism in North American whitefish species pairs (Bernatchez, 2004; Bernatchez & Dodson, 1990). Despite different divergence times at secondary contact (North America: 60,000 years, Jacobsen et al., 2012; Alpine whitefish: less than 15,000 years), the degree of RI upon secondary contact is similarly high in the two regions (*F*_ST_ North America 0.008–0.22 (Gagnaire, Pavey, Normandeau, & Bernatchez, 2013); Thun 0.08–0.21; Maggiore 0.19, Como 0.12 (Hudson et al., 2008)). The maintenance of RI of the introduced species in Lake Thun seems especially remarkable considering the high number of native whitefish species with which it now coexists.

Many studies suggest that abiotic habitat heterogeneity is crucial for the maintenance of RI between artificially stocked salmonids and their native relatives by providing opportunities for premating isolation (Dagani, 2012; Englbrecht, Schliewen, & Tautz, 2002; Marie, Bernatchez, & Garant, 2012; Winkler, Pamminger‐Lahnsteiner, Wanzenböck, & Weiss, 2011). This likely applies to Lake Thun and to those Southern Alpine lakes that now have two sympatric introduced whitefish species: They all remained well oxygenated at their greatest depths during their rather mild eutrophication (Salmaso & Mosello, 2010; Vonlanthen et al., 2012), and in Lake Thun, the introduced species shows least genetic intermediacy with native species having different spawning depth and times (*C. albellus*,* C*. sp. “Felchen”, Figure 2). It is not clear whether the indications of hybridization between *C*. sp. “Albock” and native species spawning at the same depth in winter (Figures 1 and 2) are due to natural hybridization. The spawning fishery takes place at exactly this time of the year, and inadvertent crossing in the hatchery may contribute to admixture among species.

Theory predicts that the maintenance of RI in sympatry is easier than its evolution in sympatry. For example, theoretical models find that RI between two allopatrically evolved species can be maintained in sympatry by moderate assortative mating (Kirkpatrick & Ravigné, 2002) or disruptive selection (Flaxman, Walchoder, Feder, & Nosil, 2014), whereas for the same parameter values, sympatric speciation cannot occur. In this context, it is interesting that we find a relatively high *F*_ST_ (0.12) despite a lack of significant differentiation in spawning depth or time between the introduced species and the native *C*. sp. “Balchen1” in Lake Thun, while all native species are significantly differentiated from each other in spawning time, depth, or both (Supporting Information Tables S7, S8). Moreover, our data are consistent with the idea that sympatric speciation requires strong coupling between RI and ecology, while allopatric speciation does not: *F*_ST_ is strongly correlated with differentiation in spawning depth and gill raker numbers across the four species from the spawning depth gradient, and less so for the introduced species and the presumably allopatrically evolved *C. alpinus* (Figure 3).

Finally, our data suggest that resources are differently partitioned between native species compared to between native and introduced species. Native species differ strongly in gill raker numbers and less so in water depth of feeding grounds, whereas the introduced species *C*. sp. “Albock” significantly differed in water depth of feeding grounds from most sympatric native species (Supporting Information Table S16). This is due to its restriction to surface waters, whereas most native species have greater depth ranges (Supporting Information Figure S7b). The exception is *C*. sp. “Balchen1”, which co‐occurs with the introduced species, but has very different gill raker numbers. Our results speak to the importance of multidimensional niche differentiation for the buildup of sympatric species diversity within a lineage, and also to constraints to evolve such multidimensional niche differentiation in sympatry. We note that the persistence of an introduced whitefish species in Lake Thun (and in Lake Lucerne, Hudson et al., 2016) suggests the existence of unsaturated niche space even in large whitefish radiations and that carrying capacity for whitefish species richness might not be reached by intralacustrine speciation alone (Supporting Information Appendix S12). Diversification in this case may be limited by constraints to speciation rather than by factors limiting the coexistence of ecologically divergent species.

In this study, we describe patterns of genetic divergence between all species of the largest known Alpine whitefish radiation, consisting of six sympatric species, including one previously unknown species. High species richness is attained through both sympatric ecological speciation along an environmental gradient and allopatric speciation. The maintenance of RI between a recently introduced species and all native radiation members illustrates a clear case of postglacial allopatric speciation among whitefish. Consistent with predictions from speciation theory, we find that reproductive and ecological niche differentiation are strongly correlated among species of sympatric, but not between species of allopatric origin. Overall, our study highlights the importance of multidimensional ecological and reproductive niche partitioning for evolutionary community assembly through adaptive radiation as well as the importance of multiple distinct modes of speciation for generating high local species richness.

## DATA ACCESSIBILITY

Data (sampling locations, morphological and ecological data and microsatellite genotypes) from this manuscript is publically available in the Dryad database (https://doi.org/10.5061/dryad.k183ft7).

## CONFLICT OF INTEREST

None.

## AUTHOR CONTRIBUTIONS

CD, PV, and DB carried out fieldwork and generated genetic and phenotypic data; CD analyzed the data with input from OS and CEW; CD and OS wrote the manuscript with the help of CEW; all authors commented on previous drafts of the manuscript.
