# Supplementary material for: Rapid buildup of sympatric species diversity in Alpine whitefish
Source: Ecol Evol. 2018 Aug 29;8(18):9398–412. doi: 10.1002/ece3.4375 (PMC6194267; doi:10.1002/ece3.4375)
Supplement: Supplementary file 1 [file ECE3-8-9398-s001.pdf]

Electronic supplementary.

Rapid build up of species diversity in Alpine whitefish

Appendix S1: methods. Genotyping assessment
To verify that genotyping was consistent across different scorers and sequencing machines, we newly extracted and genotyped 90 samples from Bittner (2009), and 32 samples from Vonlanthen (2009). For six individuals repeated from Bittner (2009), it was not clear whether the same individual was repeated, since repeated genotypes did not match genotypes from the previous study (≥3 markers differed). These individuals were conservatively excluded from our dataset. After excluding these individuals, 80 out of 84 repeated individuals agreed in genotype at all markers. For Vonlanthen, three of 32 repeated individuals showed genotype mismatch at one marker. To test whether scoring differed between scorers, two persons (CD and DB) scored the same 192 samples. Genotypes agreed between scorers in > 99.5% of all cases.

Appendix S2: methods. Testing for intraspecific genetic structure.
To explore intraspecific genetic structure within lakes, we performed Mantel tests based on individual data for each genetic cluster within each lake. We related individual genetic distances to individual distances in GRN, spawning depth, spawning time, habitat depth (benthic and pelagic separately) and geographic distances of spawning sites using the function "mantel" of the R-package "vegan" (Oksanen *et al.*, 2016). Multilocus individual genetic distances (â) (Rousset, 2000) were calculated in SPAGeDI v. 1.5a (Hardy and Vekemans, 2002). We adjusted p-values for multiple testing using Holm's method (Holm, 1979). To focus on variation within species and exclude potential misassignments or hybrids, we only used clearly assigned individuals (highest assignment proportion > 0.7) from contemporary samplings.

Appendix S3: methods. Testing for genetic clusters vs. continuous genetic variation along an environmental gradient.
Within each lake, we tested whether genetic variation across the four clusters found in both lakes (*C*. sp. "Balchen1", *C*. sp. "Balchen2", *C*. sp. "Felchen", *C. albellus*) could be explained by isolation by spawning depth or isolation by spawning time or isolation by distance or isolation by adaptation alone. We performed partial Mantel tests of individual genetic distance and cluster membership (a model matrix with 1 for individuals belonging to the same cluster, and 0 for different clusters) while correcting for spawning depth, spawning time, geographic or GRN differences. Since spawning depth explained more genetic variation than the other variables, we also took residuals of Mantel tests between genetic distance and spawning depth using the function "multi.mantel" of the R package "phytools" (Revell, 2012), and used those residuals in partial Mantel tests with cluster membership while correcting for either spawning time, geography or gill raker numbers.
To correct for three variables together, we first took residuals of Mantel tests between genetic distance and spawning depth using the function "multi.mantel" of the R package "phytools", then we took again residuals of Mantel tests between this residual genetic distance and geographic distance, and used those residuals in partial Mantel tests with cluster membership while correcting for GRN or spawning time differences. To correct for all four variables together, we took residuals from the previous residual genetic distance (genetic distance against spawning depth and geography) and GRN, and finally used those residuals in partial Mantel tests with cluster membership while correcting for spawning time. For Lake Thun, we only included individuals whose sum of assignment likelihoods was >0.85 for those four clusters.

Appendix S4. Results. Qualitative differences in depth range among sympatric species.
In Lake Brienz (Figure S7a), all *C.* sp. "Balchen1" were caught in the shallow littoral zone (< 11m). *C.* sp. "Balchen2" was absent from the littoral zone but occurred at all depths in all other lake habitats. *C.* sp. "Felchen" and *C. albellus* were common in all lake zones. In Lake Thun (Figure S7b), all *C.* sp. "Balchen1" and *C.* sp. "Albock" were caught at rather shallow depths (max. 27m and 34m, respectively) in both littoral and pelagic zones. *C. alpinus* was caught at all depths of the profundal benthic zone (15-207m). *C.* sp. "Balchen2", *C.* sp. "Felchen" and *C. albellus* were caught at all depths, but most rarely in the littoral zone.

Appendix S5. Results. Morphological and reproductive differences in the littoral species between lakes.
We found that the littoral spawning species "Balchen1" spawns earlier in Lake Brienz than in Lake Thun (Figure 1). This was already the case 125 years ago, as reported by Fatio (Fatio, 1890). One reason could be that the cold water temperatures, which are necessary for egg development of whitefish, are reached earlier in colder Lake Brienz than in Lake Thun. Such temporal reproductive isolation may have allowed these two populations to diverge even before the lakes were completely disconnected, and may have facilitated the evolution of weak differentiation in GRN. Another factor that could contribute to the between-lake divergence of this species is hatchery breeding of this and other species in Lake Thun, which occurred to a lesser extent in Lake Brienz (Figure S15). This could cause stronger admixture (despite efforts of fisheries mangers to breed "Balchen" and "Albock" separately), fisheries-induced selection and selection for hatchery-adapted genotypes in Lake Thun. Finally, "Balchen1" is the rarest whitefish species in both lakes (Figure 1), and its presumably small effective population size may lead to faster divergence by drift since the separation of the two lakes than for species with higher abundance and larger effective population sizes.
To assess the contribution of stocked fish to the whitefish community in the lake and to estimate the degree of admixture attributable to the fertilization process in the hatchery, mark-recapture studies could be conducted.

Appendix S6: Results. Origin of the introduced species.
Previous studies found signs of introgression of whitefish from Lake Constance into Lake Thun (Douglas *et al.*, 1999; Douglas and Brunner, 2002; Hudson *et al.*, 2011; Hudson *et al.*, 2016), however none of these studies identified which species from Lake Constance the introgression derived from (Hudson *et al.*, 2016). Lake Constance harbored five whitefish species (Steinmann, 1950), including two pelagic species (*C. macrophthalmus*, *C. wartmanni*), a littoral species (*C. arenicolus)*, a generalist species (C. sp. "Weissfelchen") and a profundal species (*C. gutturosus*), which is extinct today (Vonlanthen *et al.*, 2012). We found that historical reports document that whitefish from Lake Constance were repeatedly introduced into Lake Thun (1888 40'000 alevins, Fatio, 1890; 1934 700'000 alevins, Archiv Oberländischer Fischereiverein, Interlaken, Supplementary Figure S14), and also once in Lake Brienz (1892 39'000 alevins, Heuscher, 1901). In these reports, the introduced species was always stated to be "Blaufelchen" from Lake Constance, i.e. *C. wartmanni*. In contrast, F_{ST} comparisons of neutral microsatellites (this study), suggest that the introduced species of Lake Thun (today's "Albock") is most closely related to *C. macrophthalmus* (F_{ST} =0.028), and not to *C. wartmanni* (F_{ST} =0.110).

To further assess the introduction of whitefish from Lake Constance into Lake Thun, we identified private alleles among lake Constance whitefish species using microsatellite data from individuals collected during the pre-eutrophication period of this lake (Vonlanthen *et al.*, 2012), and we explored the occurrence of these alleles in Lake Thun. For our question, we

considered those private alleles from Lake Constance species to be informative that were found in Thun, but not in Brienz. This is because if one of the Constance private alleles is found in Thun and in Brienz, it is possible that that the allele independently evolved within this lake system, rather than being derived from stocking. Given that Lake Brienz was also at least once stocked with whitefish from Lake Constance (Heuscher, 1901), although to a much lesser extent than Lake Thun, it is possible that private alleles could have gotten into both lakes through stocking. Our test is conservative in this regard.

For each species from Lake Constance, we calculated the proportion of private alleles that fulfilled this criterion (present in Thun, absent in Brienz). We found one of two private alleles for *C. wartmanni* (50%), four of 16 for *C. macrophthalmus* (25%) and one of eight for *C. gutturosus* (12.5%) that fulfilled this criterion (Table S19) (No private alleles were found for *C. arenicolus*). These patterns are consistent with the introduction of any or all of the pelagic whitefish species from Lake Constance into Lake Thun, and thus does not identify a single most likely introduced species.

It is possible that the founder event associated with the introduction and/or admixture with native species of Lake Thun changed allele frequencies of the introduced species, which makes inference of the original species identity difficult when using few microsatellites. Furthermore, misidentification of introduced whitefish from Lake Constance is possible, as *C. wartmanni* and *C. macrophthalmus* are both pelagic fishes, and are phenotypically similar and both spawn in winter. Hence, we cannot conclusively say which species today's "Albock" from Lake Thun derives from, but both historical reports and our genetic data are most consistent with it being either one or both of these pelagic species from Lake Constance. More detailed genomic analyses would be necessary to resolve which species from Lake Constance and also from Lake Thun contribute to today's "Albock" from Lake Thun.

Appendix S7. Discussion. Taxonomic considerations
Three of the four species that we found in this study in Lake Brienz were previously known: one is taxonomically described (*C. albellus* Fatio 1890; revisions by Kottelat, 1997) and two are historically documented (Fatio, 1890) and well-known by local fishermen (*C.* sp. "Felchen" and *C.* sp. "Balchen"). Because the fourth, previously unknown species is genetically and phenotypically similar to the known *C.* sp. "Felchen" and *C.* sp. "Balchen", we call it here *C. sp.* "Balchen2". To our knowledge, genetic substructure within "Balchen" has not been previously reported. But remarkably, Fatio (1890) and Steinmann (1950) already mentioned two different types of *C. fatioi*, of which one might correspond to one of the "Balchen" clusters from this study.

C. alpinus Fatio 1885 ("Kropfer"), is a native endemic to Lake Thun and is taxonomically described (Fatio, 1885; revisions by Kottelat, 1997), whereas *C.* sp. "Albock", was introduced from Lake Constance, and is not taxonomically described. Note that despite its local name "Albock" being the same as that of native *C. fatioi*, it is certainly not this native species. It is unclear whether the taxonomically described *C. fatioi* Kottelat 1997 (Kottelat, 1997) corresponds to *C.* sp. "Balchen 2", *C.* sp. "Felchen" or something else (e.g. the whitefish species mentioned by Fatio (1890) that historically migrated between the two lakes, whose migration is now impossible due to the completion of the water gates in 1856.)

In Lake Brienz, nine individuals (7 contemporary, 2 historical scale samples) had major genetic assignment proportions for *C. alpinus,* the profundal whitefish species only known from Lake Thun (Figure S9). However, all seven contemporary genetic *C. alpinus* fish from Lake Brienz were sampled in shallow waters (Figure S6) and they had very high GRN (40-

42). This is both very atypical based on what is known from this species from Lake Thun, and we consider it unlikely that these fish are actually corresponding to *C. alpinus*. Such a mismatch could result from extensive introgression of *C. alpinus* into remaining whitefish species of Lake Brienz, as has been suggested for the very similar case in Lake Constance where the profundal species (*C. gutturosus*) is extinct today, and the few individuals from contemporary samplings that are genetically assigned to *C. gutturosus* do not phenotypically correspond to what is known from this species (Vonlanthen *et al.*, 2012).

The reasons for the absence of *C. alpinus* in Lake Brienz despite its natural presence in Lake Thun and the long shared history of the lakes can only be speculated. Higher turbidity, lower temperatures and steeper bathymetric slopes resulting in lower amounts of benthic habitat of intermediate depth in Brienz may not provide the niche requirements of the profundal benthic species, so that it never occurred there. Alternatively, *C. alpinus* might have been originally present in Lake Brienz, but went extinct after the separation of the two lakes.

Appendix S8. Discussion. Challenges and limitations to assessing sympatric whitefish species diversity.
Both the choice of genetic markers and sampling design have the potential to bias the number of species and the genetic structure among species we can recover in our dataset.

The six species we identified in this study should be considered as a minimum estimate for the actual whitefish species diversity present in Lakes Brienz and Thun. With 10 neutral markers and no prior grouping based on phenotypes, we are limited to detect common and clearly reproductively isolated groups, and we miss rare and/or only weakly differentiated whitefish species. For example, while RADseq data show very clear separation of three whitefish species from Lakes Walen and Zürich (max. F_{ST}=0.11, min. F_{ST}=0.03, unpublished data, Feulner *et al.*), our 10 microsatellites could not resolve any of these species using the same samples (N=20 per species) with the program Structure. However, F_{ST}s among groups were the same for both types of data (data not shown). We therefore suggest combining genomic data with ecological and morphological data to identify such rare populations and to resolve genetic structure among them.

It is well known that discontinuous sampling of a continuous distribution can produce discrete genetic clusters in structure analyses (e.g. Serre, 2004; Rosenberg *et al.*, 2005). In our study, we can reproduce a more discontinuous distribution of genetic variation when considering only samples from targeted spawning fishing, for example, using whitefish from Lake Brienz from Bittner (2009) (Figure S9). Vice versa, when relying on samples from random, quantitative fishing alone, the unequal abundance of genetic variation makes detection of population structure using clustering programs such as Structure (Pritchard *et al.*, 2000) very difficult. Furthermore, some genotype combinations that are not uncommon in the targeted sampling are completely absent from the random sampling (e.g. genetic intermediates between *C.* sp. “Balchen1” and *C.* sp. “Balchen2”(Figure S9)), which gives a misleading picture of the actual structure in the whitefish community. Hence, the two types of samplings are complementary, and both are needed to assess whitefish diversity and its genetic structure in these lakes.

Appendix S9. Discussion. Multiple dimensions of niche partitioning contribute to RI and coexistence in sympatry.
In both lakes, we found significant differentiation in spawning depth and/or spawning time among all native species (Figure 1, Table S7, S8), whereby the former was generally stronger

and predicted the degree of genetic differentiation better than the latter. This suggests a greater importance of spawning depth than of spawning time for maintaining RI among sympatric Alpine whitefish species, consistent with studies of other Alpine lakes (Vonlanthen *et al.*, 2009; Ingram *et al.*, 2012; Hudson *et al.*, 2016). Spawning depth differentiation also plays a major role for RI in other young species radiations of fish, such as haplochromine cichlid fishes in Lake Victoria (Seehausen *et al.*, 2008) and benthic-limnetic stickleback species pairs in Canada (Hatfield and Ptolemy, 2001).
The parallelism of the two dimensions of reproductive niche (Figure 1) likely strengthens total RI among species, as indicated by comparisons to Alpine whitefish radiations without spawning time segregation. In Lakes Thun and Brienz, the four species from the spawning depth gradient do not only spawn at different depth, but also at different times of the year, whereas the less strongly differentiated species from Lake Lucerne's and Neuchâtel's spawning depth gradient all spawn in winter and do not differ in spawning time anymore (Hudson *et al.*, 2016; Vonlanthen *et al.*, 2009). Historically, stronger temporal spawning segregation also occurred among Lake Lucerne whitefish, with *C. zugensis* spawning either in summer or in January and *C. nobilis* in summer (Steinmann, 1950). Lake eutrophication, which was stronger in Lakes Lucerne and Neuchâtel than in Thun and Brienz, caused low oxygen concentrations at great depth in summer, which could have favored individuals that spawn later in the year, which increased the chances for admixture with winter spawning species. Eutrophication could therefore have increased gene flow among species by contracting both the spatial and the temporal spawning gradient.

Our finding that differentiation in gill raker numbers explains residual genetic differentiation not explained by spawning depth differentiation (Figure 3e) could indicate that pre-zygotic spawning segregation and post-zygotic divergent natural selection complement each other to maintain RI among sympatric whitefish species.

Appendix S10. Discussion. More interspecific gene flow in Lake Thun than in Lake Brienz.
Although species are fewer in Lake Brienz than in Lake Thun, genetic distinctiveness is higher among species of Lake Brienz. We find several lines of evidence that suggest higher rates of gene flow between species in Lake Thun than in Lake Brienz. Lake Thun has more individuals with intermediate assignment likelihoods (Table S6), the four species that occur in both lakes show weaker genetic differentiation between them in Lake Thun (Table 1), and in genetic PCA, species of Lake Thun show more overlap in genotype space than species of Lake Brienz (Figure 1). Finally, the relationship between genetic assignment and GRN between closely related species is weaker in Lake Thun than in Lake Brienz (Figure S3). One explanation for higher rates of interspecific gene flow in Lake Thun could be that higher species numbers lead to more opportunities for hybridization. However, when considering individuals having minor genetic contributions from the two species unique to Lake Thun, *C. alpinus* and *C.* sp. "Albock" (<0.2), F_{ST}s still remain clearly lower in Lake Thun than in Brienz (Table S20). Alternative explanations for weaker RI are stronger anthropogenic influences in Lake Thun than in Brienz (e.g. eutrophication and stocking, Vonlanthen *et al.*, 2012; Figure S16). Disruptive selection could also just naturally be stronger in Lake Brienz. Finally, if effective population sizes of species were consistently lower in Lake Brienz than in Lake Thun, we would expect the dynamics of incomplete lineage sorting to lead to shorter coalescent times and the appearance of fewer intermediate genotypes among species of Lake Brienz.

Appendix S11. Discussion. Increased abundance of the introduced species.
A comparison between genetic data from the 1950s-1970s and from 2004-2014 indicates that the importance of the introduced species changed over time for professional fisheries. Genetic

data from the 1970s suggest that the introduced species was absent in catches of professional fishermen in Lake Thun (Figure S12). Contrary, after 2000, it was common in spawning fishery catches of commercial fishermen (qualitative data only) (Figure S14) and it made up 8.4% of individual whitefish in the catches of habitat stratified random fishing (Figure S5, S14). Whether the introduced species indeed increased in abundance only during the last third of the 20th century, or whether it was already common before, but not present in our samples from 1950-1970, is not clear from our data. However, if the former was the case, it could be hypothesized that its rise in abundance was mediated by the peak of mild eutrophication in Lake Thun in the 70s/80s.

Appendix S12. Discussion. The buildup of local whitefish species diversity is limited by constraints to speciation

The establishment and persistence of an introduced whitefish species in Lake Thun (and also in Lake Lucerne, Hudson *et al.*, 2016) suggests that unsaturated niche space exists even in large whitefish radiations, and that carrying capacity for whitefish species richness might not be reached over postglacial times by intralacustrine speciation alone. Hence, speciation seems to be the limiting step for achieving high species richness in young adaptive radiations of whitefish. The extent of the ecological gradient likely determines the number of whitefish species that evolve sympatrically within a lake within a given time (Vonlanthen *et al.*, 2012). The buildup of local species richness through secondary contact between allopatrically evolved species, on the other hand, is limited by a lake's geographical isolation, and at a larger scale by the number of isolated lakes available for allopatric speciation. Hence, if isolation among lakes is strong and ecological opportunity within lakes large, the majority of species in each lake may still derive from sympatric speciation, as seems to be the case in Alpine whitefish (this study, Hudson *et al.*, 2016; Hudson *et al.*, 2011) and haplochromine cichlids (Wagner *et al.*, 2014). If connectivity among geographically isolated areas then suddenly increases after a period of isolation, be it due to human-mediated movements (whitefish this study, Hudson *et al.*, 2016; cichlids from islands Young *et al.*, 2009) or natural changes in lake levels (e.g. Lake Tanganyika), species richness in local communities may drastically increases.

Supplementary Figures

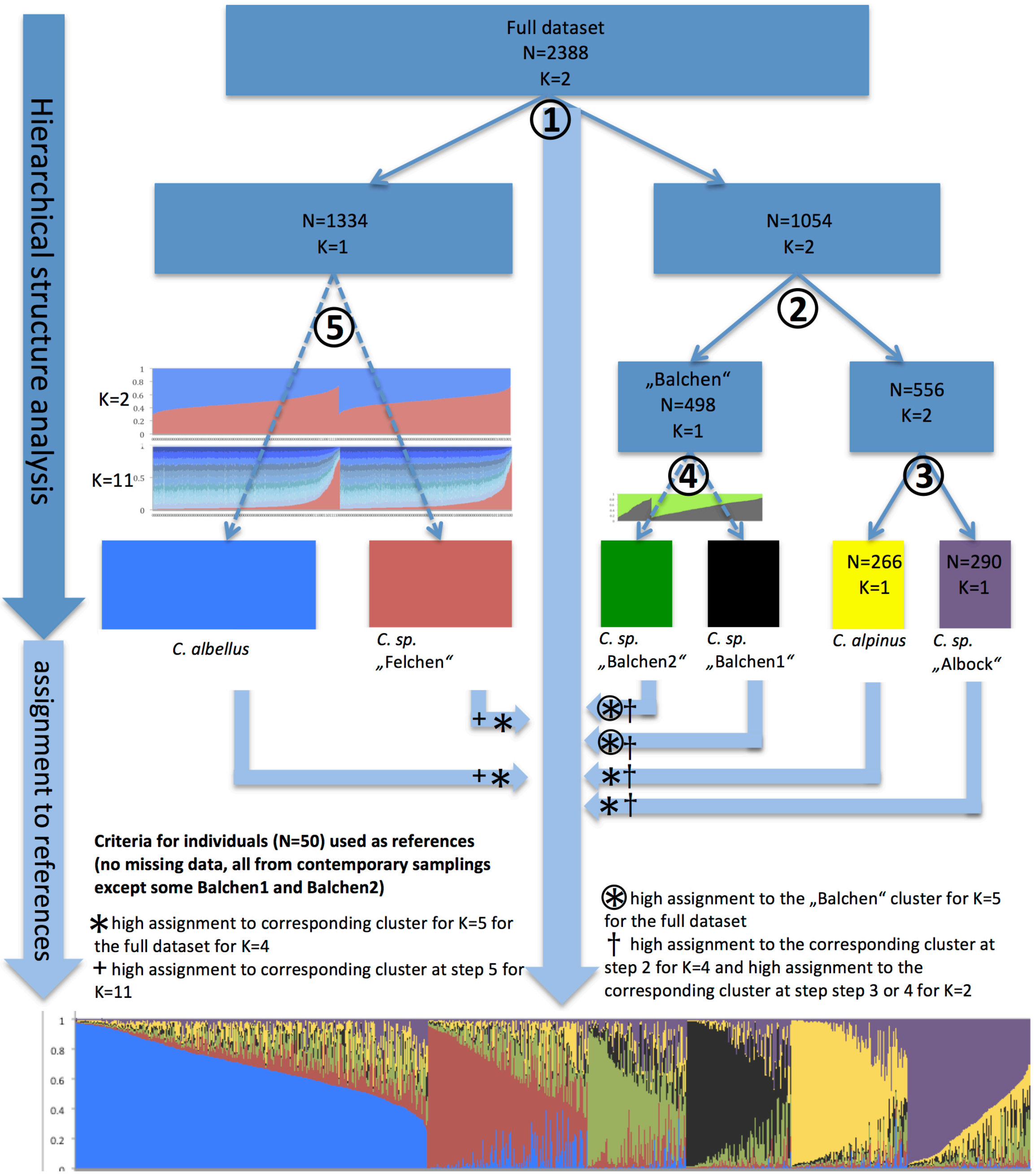

Figure S1. Schematic overview of the analysis workflow of the hierarchical structure analysis (blue) and the subsequent reference based assignment analysis (light blue). The former was used to identify genetic clusters in the full dataset, the latter to assign all individuals to the clusters identified in the hierarchical analysis. For each data subset, sample size (N) and the most likely K value are given. Hierarchical steps are labeled with circled numbers above arrows corresponding to the splits in the dataset. Solid arrows (step 1,2,3) indicate that the most likely K was >1. Dashed arrows (step 4 and 5) indicate similarly high likelihood for K=2 and K=1 and associations between genetic and ecological structure in both lakes, suggesting

the presence of two biologically meaningful genetic groups within these genetic clusters (*C*. sp. “Felchen” and *C. albellus*, and *C*. sp. “Balchen 1” and *C*. sp. “Balchen 2”, see Figure S2, S3). The bottom structure plot shows the result of the assignment analysis, wherein individuals are sorted by species (based on maximum assignment) and decreasing assignment likelihood within each species.

Analysis description: To find the most likely value of K, we conducted a hierarchical cluster analysis (Figure S1; Coulon *et al.,* 2008) using the individual-based Bayesian clustering algorithm implemented in SRUCTURE (Pritchard *et al.,* 2000). We first determined the most likely number of clusters (K) for the full dataset, then the most likely K within each of the data subsets suggested by the initial analysis, and so forth until all subsets supported a value of K=1. To determine the most likely K at each hierarchical level, we first compared LnP(D) values from runs of different Ks, as suggested by Pritchard and Wen (2003). If this method suggested K>1 was most likely, we determined the most likely K using the delta K method of Evanno *et al.* (2005) (note that the Evanno *et al.* method cannot evaluate K=1, hence this requires to also evaluate LnP(D) values). We ran Structure for K=1 to K=15 at each hierarchical level, with 10 iterations at each K value, using 100’000 burn-in steps followed by 500’000 MCMC steps to generate the posterior sample distribution. For all runs, we used the admixture and correlated allele-frequency model. We subdivided the original dataset into the K subsets suggested by the most likely K by assigning individuals to the cluster for which they had the highest assignment likelihood in the run with the highest likelihood for this K. To determine correspondence of genetic clusters to known species, we assessed how individuals from samplings targeted to known species were distributed amongst the clusters.

Because LnP(D) of K=2 and K=1 were very similar for the cluster from the hierarchical analysis corresponding to “Balchen” (Figure S2), we further explored the distribution of assignment likelihoods within this cluster for K=2. For each lake, we tested whether the two “Balchen” clusters (individuals were assigned based on their maximum assignment proportion obtained from Structure analysis on this entire cluster for K=2) differed in GRN and spawning depth using Wilcoxon tests, and we assessed the relationship between Structure assignment likelihoods to “Balchen” cluster 1 and GRN in spearman’s rank correlation tests.

The cluster derived from the hierarchical approach containing individuals from known spawning sites of *C*. sp. “Felchen” and *C. albellus* also showed very similar values of LnP(D) for K=2 and K=1 (Figure S2). Therefore, we explored genetic structure within this cluster in more detail. To test whether unequal sample sizes of *C*. sp. “Felchen” and *C. albellus* reduced Structure’s ability to distinguish the two groups, a well-known limitation of this program (Puechmaille, 2016), we performed a Structure analysis using equal numbers of mature adult individuals from three *C. albellus* and three *C*. sp. “Felchen” spawning sites (N=84 for each species) from Lake Brienz (all individuals from these 6 spawning sites were included). We ran Structure for K=1-4 with 3 replicates per K, with 100’000 burn-in steps and 50’000 MCMC steps using the admixture and correlated allele frequency model. Additionally, we assessed the relationship between Structure assignment likelihoods to *C*. sp. “Felchen” (obtained from Structure analysis on the entire *C.albellus*- *C*. sp. “Felchen” cluster for K=2) and GRN in spearman rank correlation tests within each lake. To guard against the possibility that introgression from the low GRN species *C. alpinus* confounded a potential relationship in Lake Thun (*C. alpinus* is absent from Lake Brienz), we performed this analysis by including only individuals with more than 30 gill rakers. Furthermore, we tested whether the two groups (individuals were assigned based on their maximum assignment proportion) differed in GRN and spawning depth using Wilcoxon tests.

To obtain for each individual genetic assignment proportions to the clusters inferred in the hierarchical analysis, we performed Structure assignment analyses using the clusters identified by the hierarchical analysis as reference populations (Figure S1). Genotypic data from 50 representative individuals from each of the 6 clusters identified before (named according to the known and newly recognized species they contain: *C*. sp. “Felchen”, *C. albellus*, *C*. sp. “Balchen1”, *C*. sp. “Balchen2”, *C. alpinus* and *C*. sp. “Albock”) were used to define reference populations by setting PopFlag = 1 and the corresponding population number. We therefore chose the 50 individuals with highest assignment likelihood in the corresponding clusters at each previous step in the hierarchical analysis (see text in figure for detailed criteria). For all reference populations, we only used contemporary individuals with no missing data, except for the *C*. sp. “Balchen 1” and *C*. sp. “Balchen 2” references, where we also used data from the 1950s-1970s, since these groups are rare in all samples. All remaining whitefish in the dataset (N=2088) were then assigned to the reference populations by coding them with PopFlag = 0 and population = 0, and separate analyses with subsets of individuals to assign were performed. Each of these analyses consisted of the 6*50 reference individuals plus 50 individuals to be assigned. For each of these analyses, we performed 10 replicates of K=6. We used Structure Harvester to generate input files for CLUMPP (Jakobsson and Rosenberg 2007), which we used to generate consensus percentages of assignment proportions across each of the 10 structure runs. The maximum difference in assignment likelihoods among the 10 runs was less than 0.055 for any assigned individual, and was less than 0.05 among the 420 runs for the reference individuals, indicating consistency across runs.

In Lake Brienz, nine individuals (7 contemporary, 2 historical scale samples) had major genetic assignment proportions for *C. alpinus,* the profundal whitefish species only known from Lake Thun (Figure S9). However, all seven contemporary genetic *C. alpinus* fish from Lake Brienz were sampled in shallow waters (Figure S6) and they had very high GRN (40-42). This is both very atypical based on what is known from this species from Lake Thun, and we consider it unlikely that these fish are actually corresponding to *C. alpinus*. Such a mismatch could result from extensive introgression of *C. alpinus* into remaining whitefish species of Lake Brienz, as has been suggested for the very similar case in Lake Constance where the profundal species (*C. gutturosus*) is extinct today, and the few individuals from contemporary samplings that are genetically assigned to *C. gutturosus* do not phenotypically correspond to what is known from this species (Vonlanthen *et al.,* 2012).

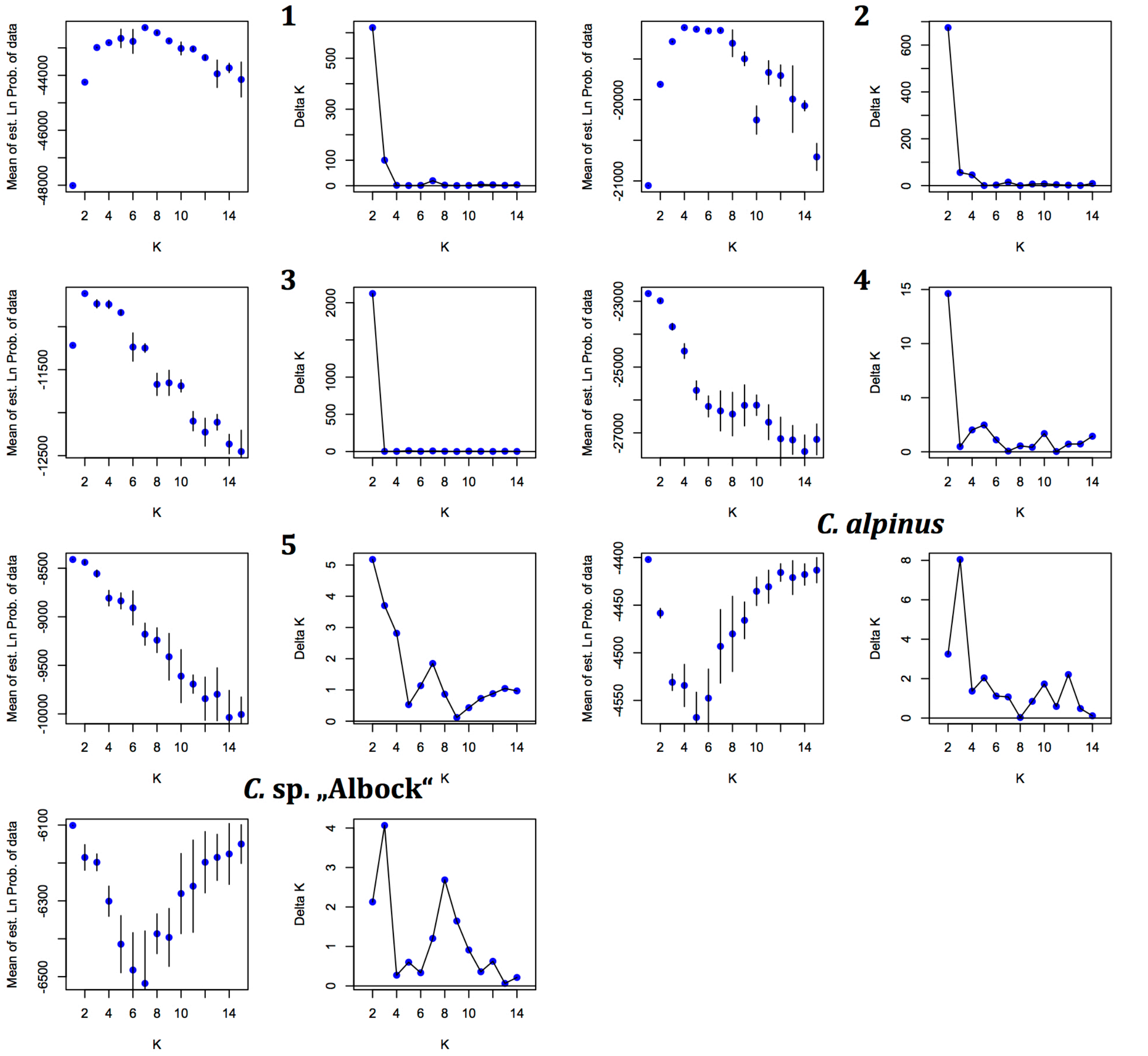

Figure S2. Mean ln probability ± standard deviations and delta K of 10 runs for K=1 to K=15 for the different clusters of the hierarchical Structure analysis. Numbers correspond to hierarchical steps labeled with circled numbers in Figure S1.

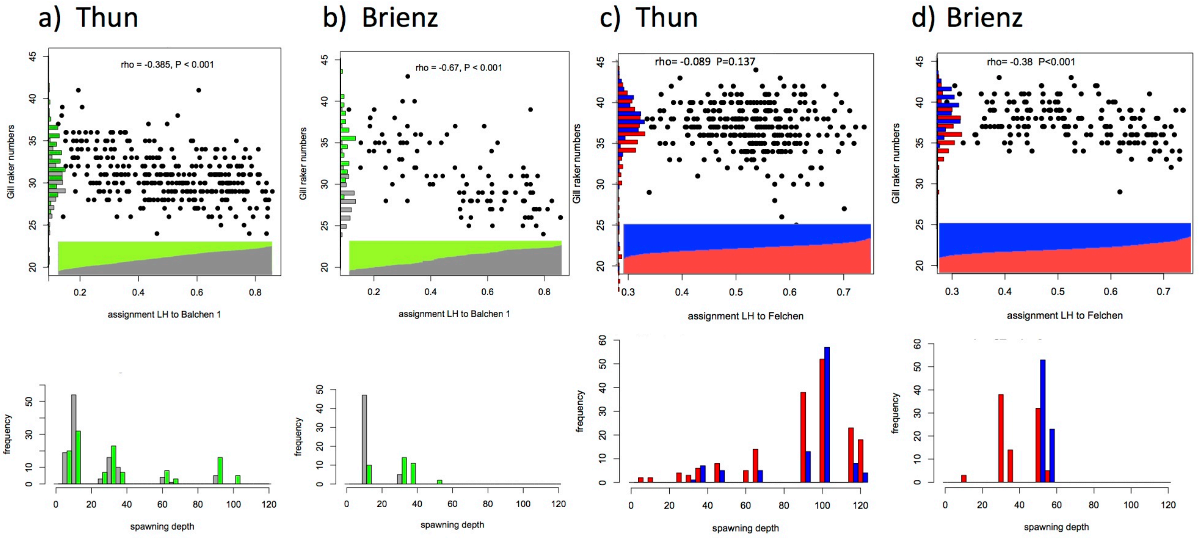

Figure S3. Genetic and ecological structure within the "Balchen" cluster (a,b) and within the *C.* sp. "Felchen"-*C. albellus* cluster in Lakes Thun (a,c) and Brienz (b,d). **a, b) top:** Genetic assignment to *C.* sp. "Balchen 1" (obtained from Structure analysis for K=2) is significantly related to the number of gill rakers in both lakes. Histograms of gill raker numbers for the two groups are shown along the y-axis (individuals are assigned based on their maximum assignment proportion obtained from Structure for K=2). Structure plots for K=2 are shown along the x-axis and are sorted by lake and increasing assignment to *C.* sp. "Balchen 1". **bottom:** *C.* sp. "Balchen 1" spawns at significantly shallower depth than *C.* sp. "Balchen 2" in both lakes. **c,d) top:** Genetic assignment to *C.* sp. "Felchen" (obtained from Structure analysis for K=2) is significantly related to gill raker numbers in Lake Brienz, but not in Lake Thun. Histograms of gill raker numbers for the two groups are shown along the y-axis (individuals are assigned based on the maximum assignment proportion obtained from Structure for K=2). Structure plots for K=2 are shown along the x-axis and are sorted by lake and increasing assignment to *C.* sp. "Felchen". **bottom:** In Lake Brienz, *C. albellus* spawns at significantly greater depth than *C.* sp. "Felchen", whereas in Lake Thun, *C. albellus* does not significantly differ in spawning depth from *C.* sp. "Felchen".

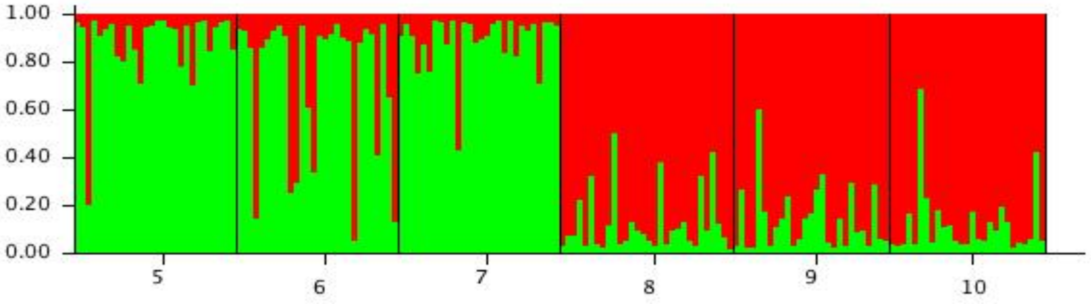

Figure S4. Structure analysis for K=2 with reproductively mature, ripe individuals from three *C. albellus* spawning sites and from three *C.* sp. "Felchen" spawning sites from Lake Brienz.

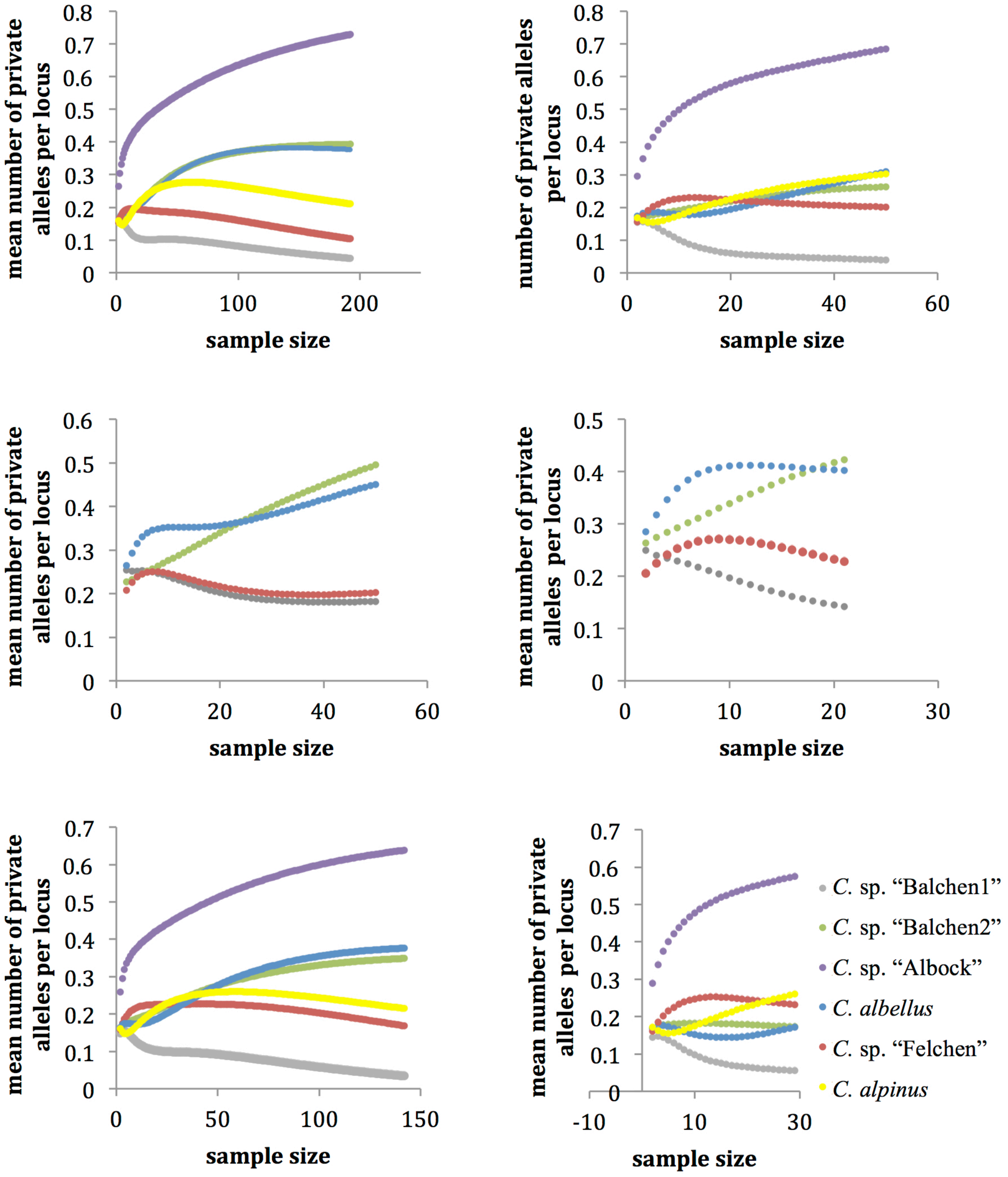

Figure S5. Private allele analyses for all whitefish species from Lakes Thun and Brienz. For each species, the mean number of private alleles per locus is plotted as a function of standardized sample size. a,c,e) show the frequency of private alleles for contemporary whitefish considering all individuals (species assignment based on maximum assignment LH in the Structure assignment analysis), whereas b,d,f) show the private allele frquencies only for clearly assigned individuals (structure assignment LH > 0.7). Panels a and b are for individuals from both lakes combined, c and d for Lake Brienz, e and f for Lake Thun.

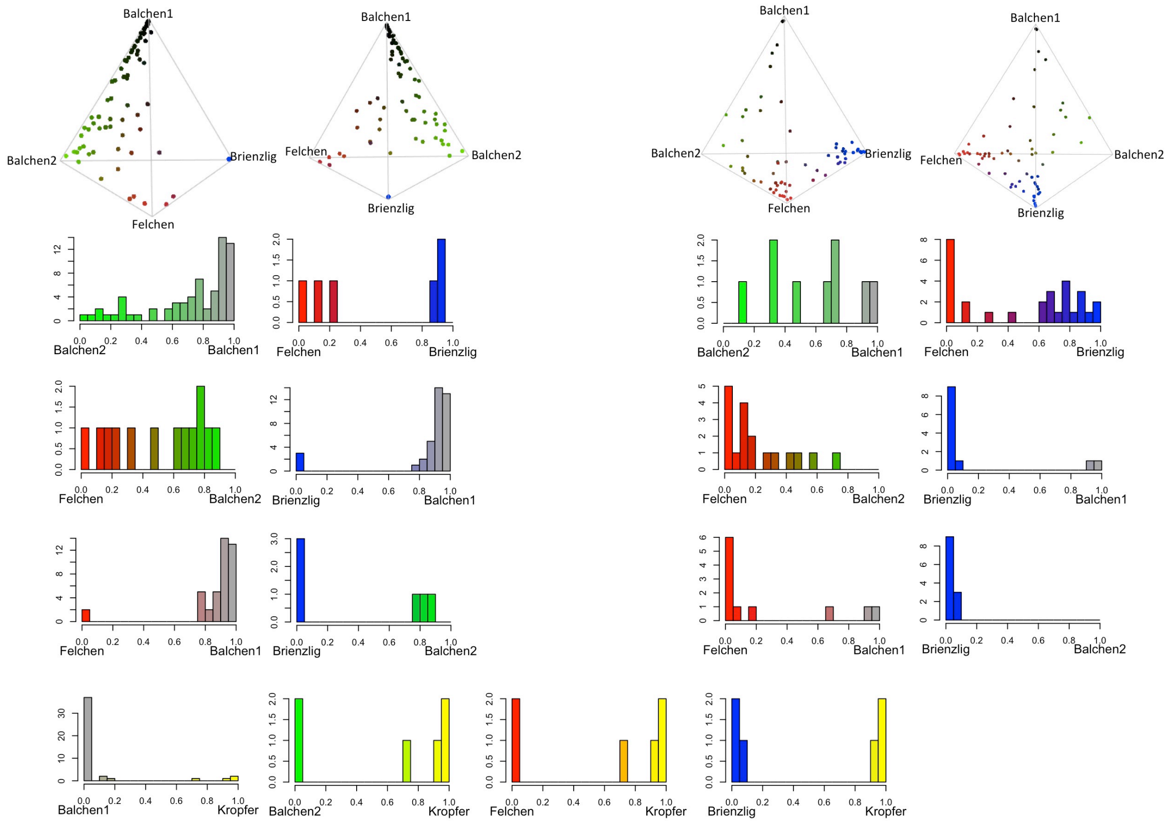

Figure S6. Genotypic distribution of the whitefish community from the 1950-70s in Lake Thun (left) and in Lake Brienz (right).

a) Tetrahedral plot show the genotypic distribution of the whitefish community in Lake Thun (left) and Lake Brienz from the 1950-70s (right). The location of an individual whitefish is determined by its Structure assignment proportions obtained from the analysis using reference populations: The corners of the tetrahedron correspond to 100% assignment to a cluster, intermediate genotypes lie within the space framed by the corners. An individual's color corresponds to its combination of assignment proportions for the different reference clusters.

b) Frequency distributions of assignment proportions from the analysis using reference populations for all pairs of genetic clusters for whitefish from Lake Thun (left and two bottom panels) and Lake Brienz (right). For each plot, only individuals that are assigned to either one of the two genetic clusters under consideration or are genetically intermediate to these and not closer to any of the other genetic clusters are used. The frequency distribution of assignment proportion of the individuals fulfilling this criterion is plotted for one of the genetic clusters under consideration (the one indicated at position 1.0). Note that the comparisons with *C. alpinus* are only shown for Lake Thun, since this species are absent in Lake Brienz.

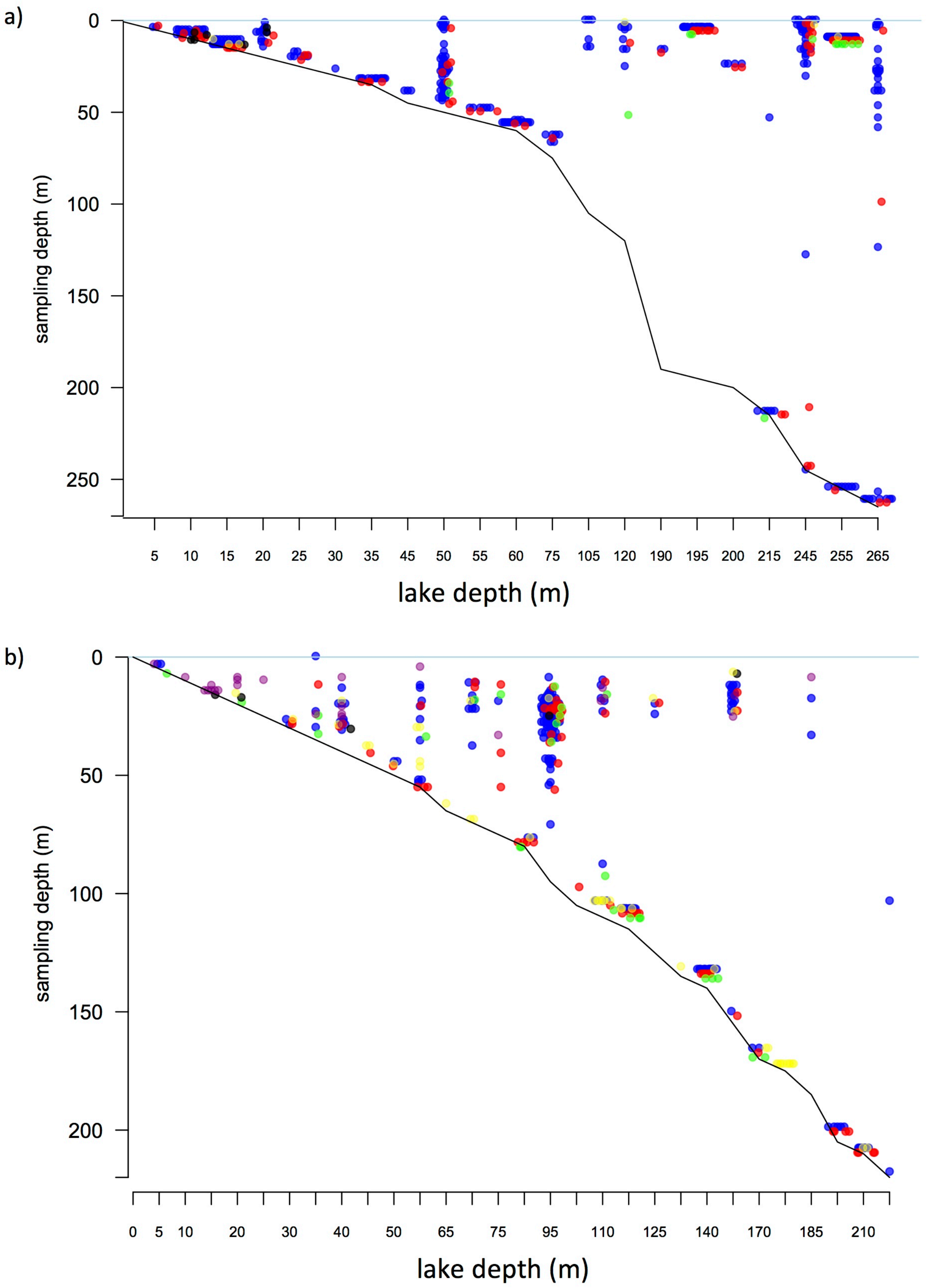

Figure S7. Spatial distribution of whitefish species as revealed by habitat stratified random fishing in Lake Brienz (a) and Lake Thun (b). Dots represent individual whitefish, which are colored by their maximum assignment to the 6 reference populations. (*C. albellus* blue; *C.* sp. "Felchen" red, *C.* sp. "Balchen 1" black; *C.* sp. "Balchen 2" green; *C.* sp. Albock" purple; *C. alpinus* yellow). We used the R package "beeswarm" on groups of 5m lake depth intervals for these graphs, and the function "jitter" (with jitter amount of 0.2 and 2 for x- and y-axes, respectively) to increase scatter among individuals for better visualization.
Black line represents the lake bottom.

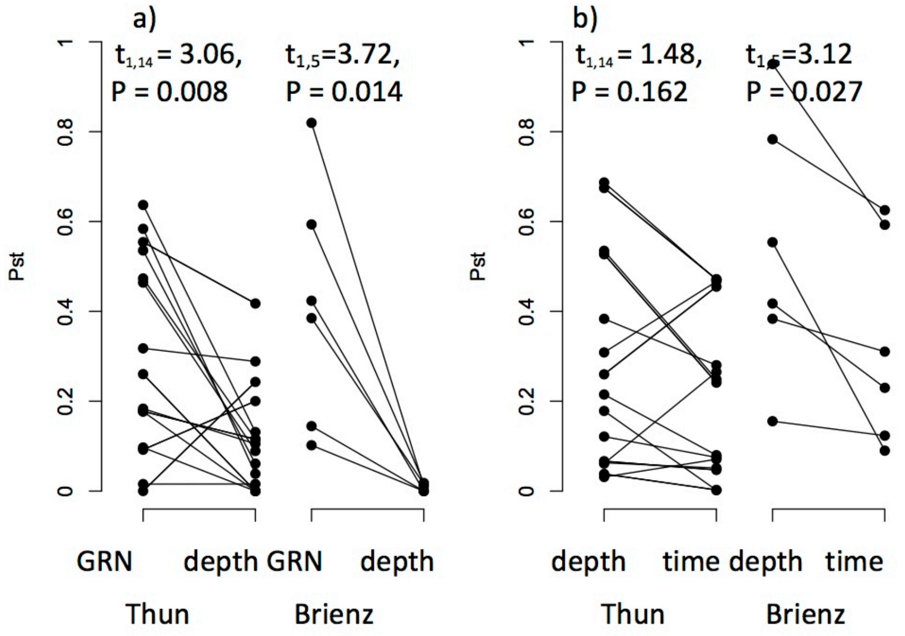

Figure S8. Comparisons between differentiation (P_{ST}) in different ecological (gill raker numbers vs. habitat depth, a) and reproductive niches (spawning depth and spawning time, b) in each lake. Individual points are pairwise comparisons between sympatric species, results of paired t-tests are indicated on top.

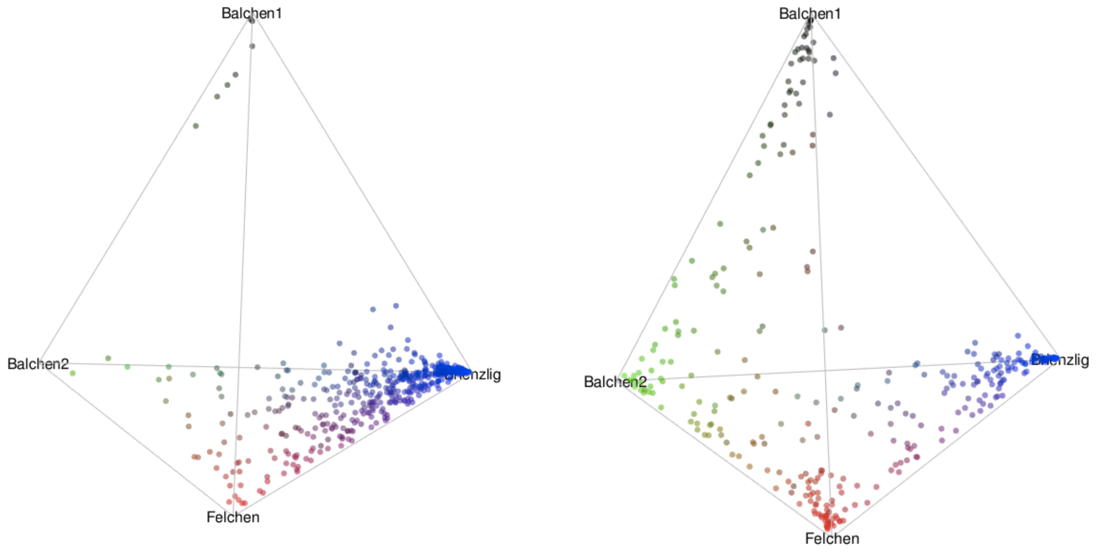

Figure S9. Habitat stratified random sampling (left) and targeted spawning samplings (right) are sampling different parts of the genotypic whitefish diversity of Lake Brienz. Intermediate genotypes between *C.* sp. "Balchen 1", *C.* sp. "Balchen 2" and *C.* sp. "Felchen" are not present in the quantitative sampling, whereas backcrosses to *C.* sp. "Balchen 2" of *C. albellus*- *C.* sp. "Balchen 2" hybrids are lacking in the spawning sampling.

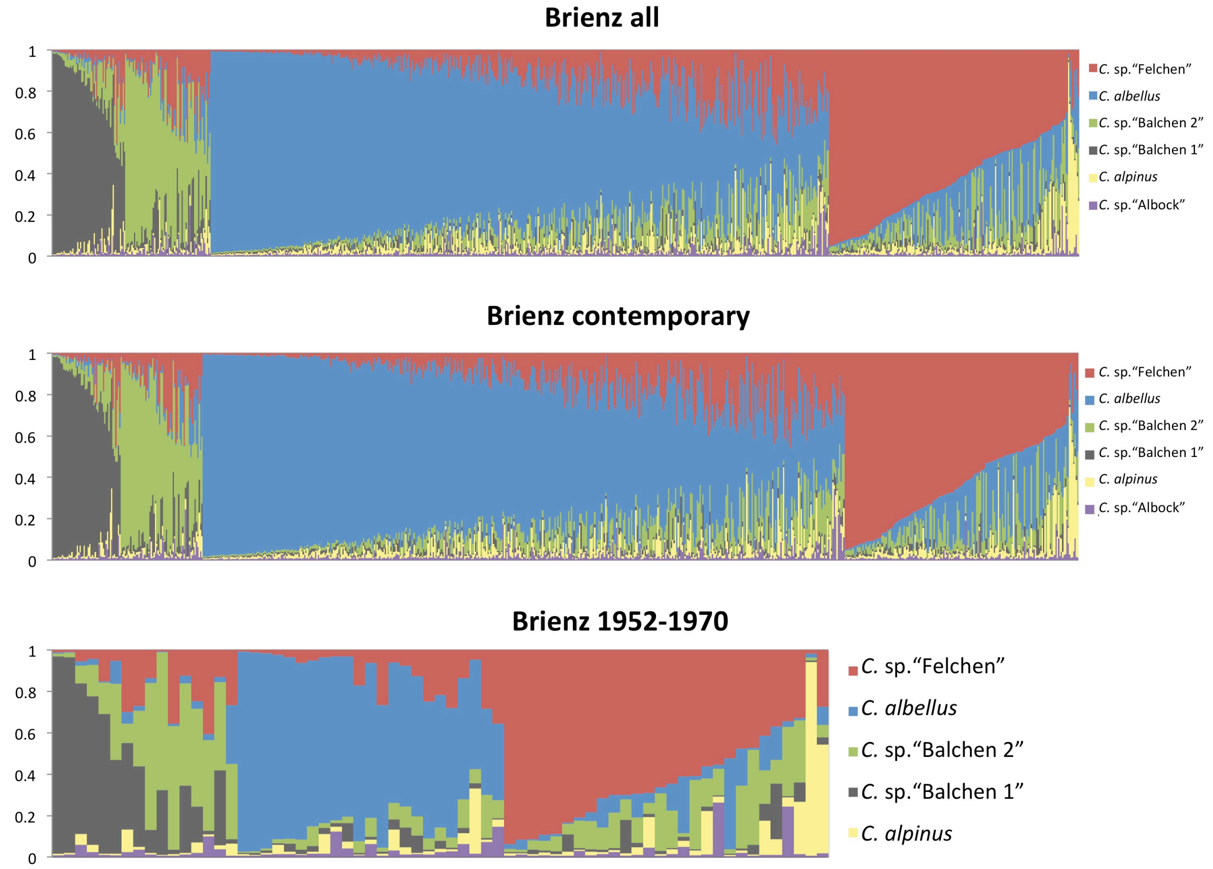

Figure S10. Results from the Structure analysis using reference populations for whitefish from Lake Brienz. The top panel shows assignment results for all samples from Lake Brienz, the middle for contemporary samples, and the bottom for historical samples. Individuals are arranged by species (determined by maximum assignment likelihood), and sorted by decreasing assignment likelihood to the corresponding species.

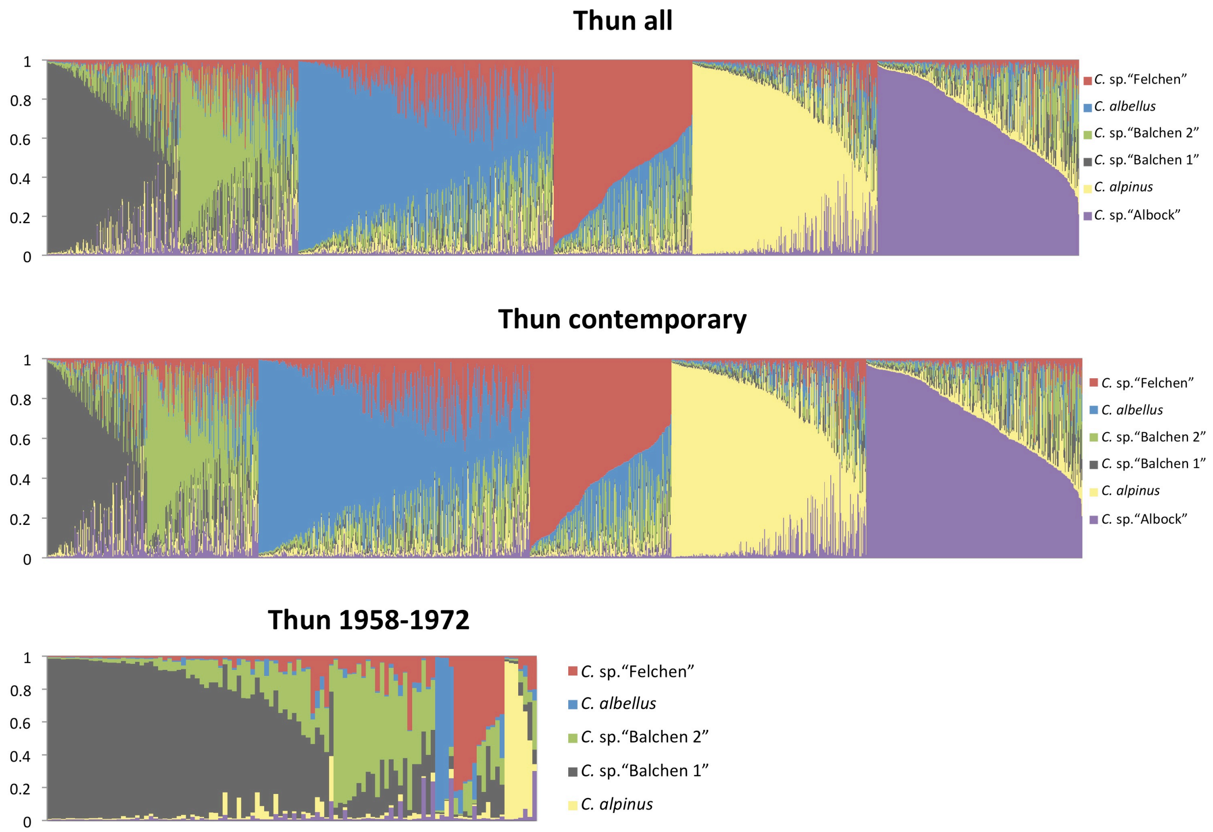

Figure S11. Results from the Structure analysis using reference populations shown for whitefish from Lake Thun. The top panel shows assignment results for all samples from Lake Thun, the middle for contemporary samples, and the bottom for historical samples. Individuals are arranged by species (determined by maximum assignment likelihood), and sorted by decreasing assignment likelihood to the corresponding species.

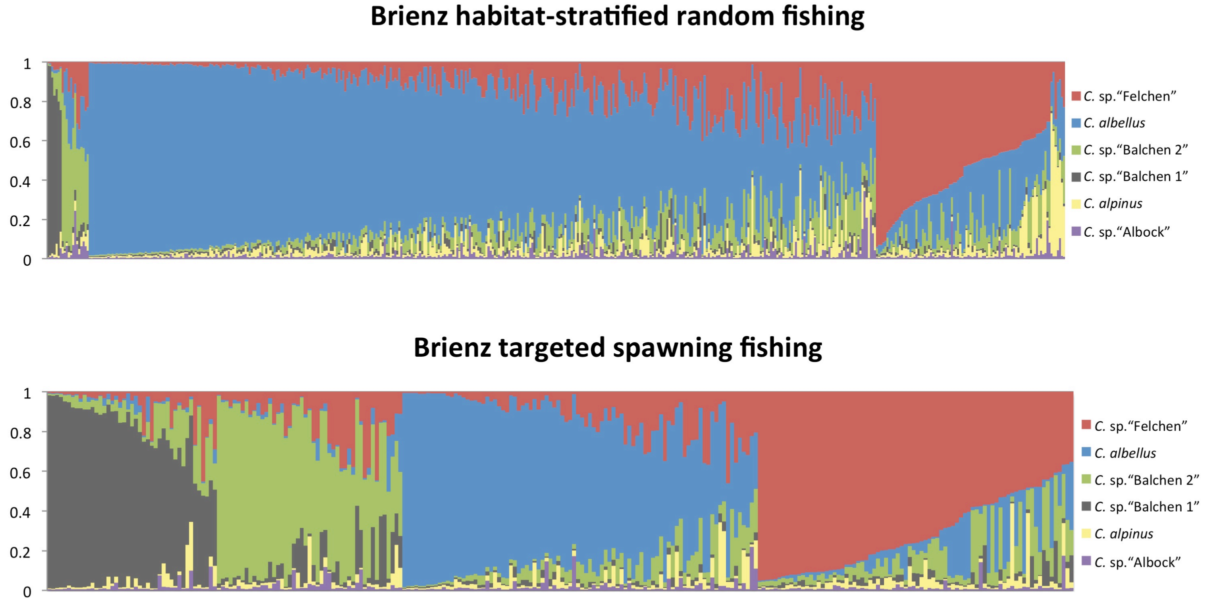

Figure S12. Results from the Structure analysis using reference populations shown for whitefish from Lake Brienz caught during the habitat stratified random sampling and the targeted sampling to known species during spawning time. Individuals are arranged by species (determined by maximum assignment likelihood), and sorted by decreasing assignment likelihood to the corresponding species.

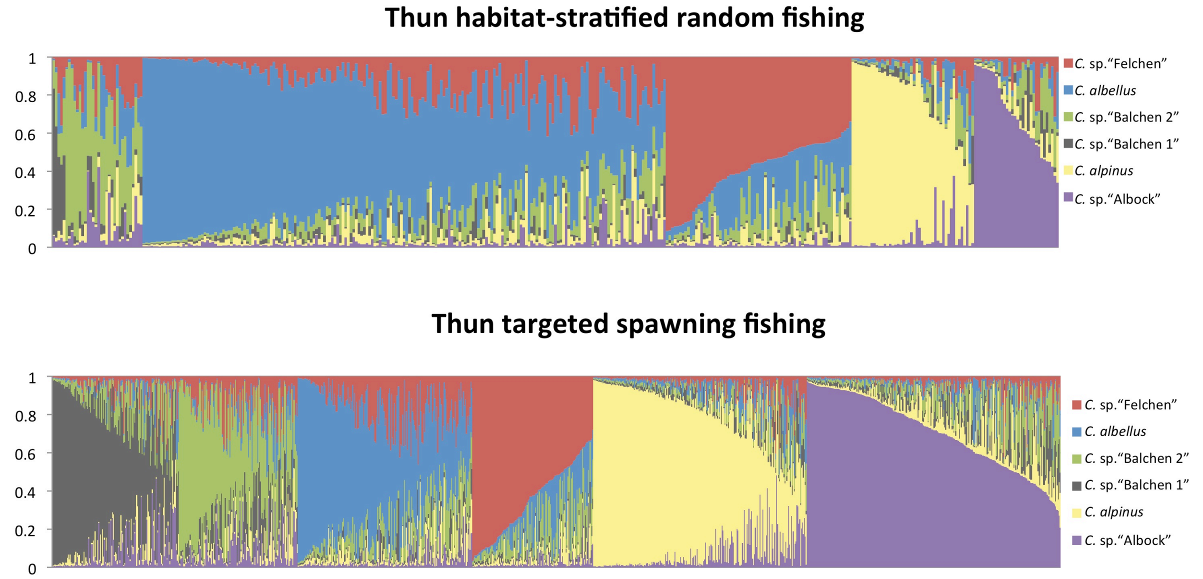

Figure S13. Results from the Structure analysis using reference populations shown for whitefish from Lake Thun caught during the habitat stratified random sampling and the targeted sampling to known species during spawning time. Individuals are arranged by species (determined by maximum assignment likelihood), and sorted by decreasing assignment likelihood to the corresponding species.

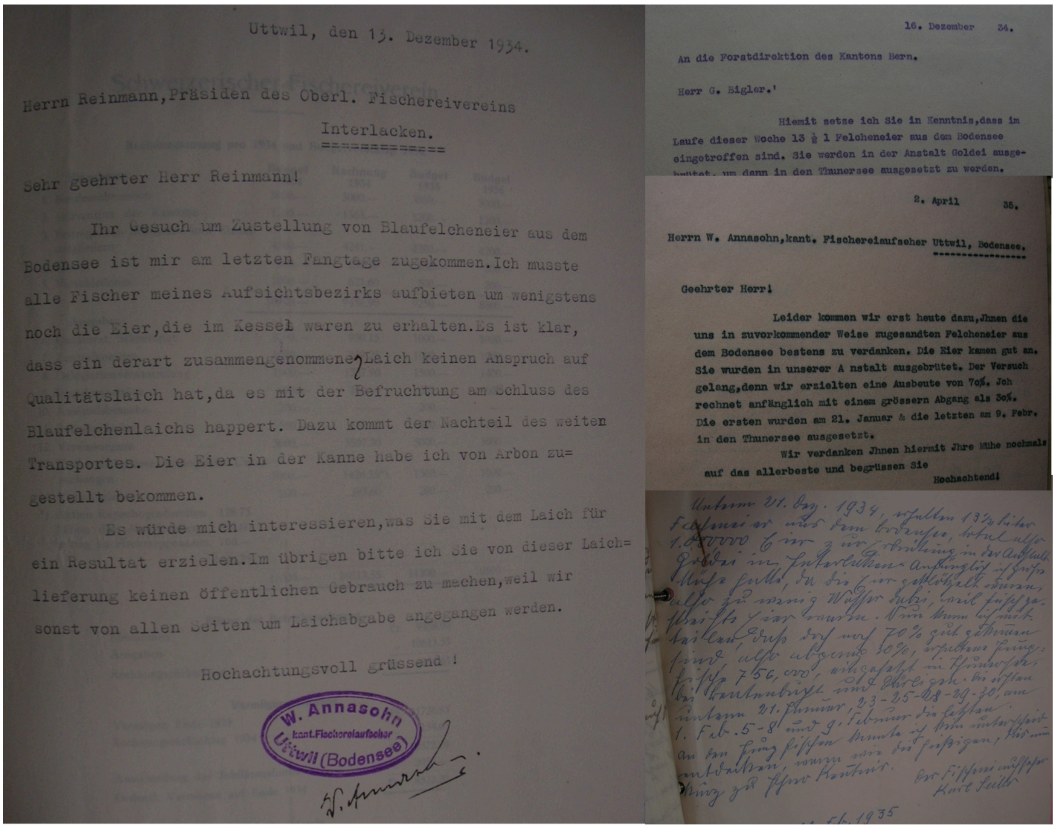

Uttwil, den 13. Dezember 1934.

Herrn Reinmann, Präsiden des Oberl. Fischereivereins

Interlacken.

Sehr geehrter Herr Reinmann!

Ihr Gesuch um Zustellung von Blaufelcheneier aus dem Bodensee ist mir am letzten Fangtage zugekommen. Ich musste alle Fischer meines Aufsichtsbezirks aufbieten um wenigstens noch die Eier, die im Kessel waren zu erhalten. Es ist klar, dass ein derart zusammengenommener Laich keinen Anspruch auf Qualitätslaich hat, da es mit der Befruchtung am Schluss des Blaufelchenlaichs happert. Dazu kommt der Nachteil des weiten Transportes. Die Eier in der Kanne habe ich von Arbon zu= gestellt bekommen.

Es würde mich interessieren, was Sie mit dem Laich für ein Resultat erzielen. Im übrigen bitte ich Sie von dieser Laich= lieferung keinen öffentlichen Gebrauch zu machen, weil wir sonst von allen Seiten um Laichabgabe angegangen werden.

Hochachtungsvoll grüssend !

W. Annasohn kant. Fischereiaufseher Uttwil (Bodensee)

W. Annasohn

16. Dezember 34.

An die Forstdirektion des Kantons Bern.

Herr G. Bigler.'

Hiemit setze ich Sie in Kenntnis, dass im Laufe dieser Woche 13 ½ l Felcheneier aus dem Bodensee eingetroffen sind. Sie werden in der Anstalt Goldei ausge- brütet, um dann in den Thunersee ausgesetzt zu werden.

2. April 35.

Herrn W. Annasohn, kant. Fischereiaufseher Uttwil, Bodensee.

Geehrter Herr!

Leider kommen wir erst heute dazu, Jhnen die uns in zuvorkommender Weise zugesandten Felcheneier aus dem Bodensee bestens zu verdanken. Die Eier kamen gut an. Sie wurden in unserer Anstalt ausgebrütet. Der Versuch gelang, denn wir erzielten eine Ausbeute von 70%. Jch rechnet anfänglich mit einem grössern Abgang als 30%. Die ersten wurden am 21. Januar & die letzten am 9. Febr. in den Thunersee ausgesetzt.

Wir verdanken Jhnen hiermit Jhre Mühe nochmals auf das allerbeste und begrüssen Sie

Hochachtend!

Unterm 21. Dez. 1934, erhalten 13½ Liter [illegible]

Figure S14. Reports from the local fishery inspector and fisheries guard documenting the introduction of Lake Constance whitefish into Lake Thun in 1935 (Archiv Oberländischer Fischereiverein, Interlaken, Switzerland).

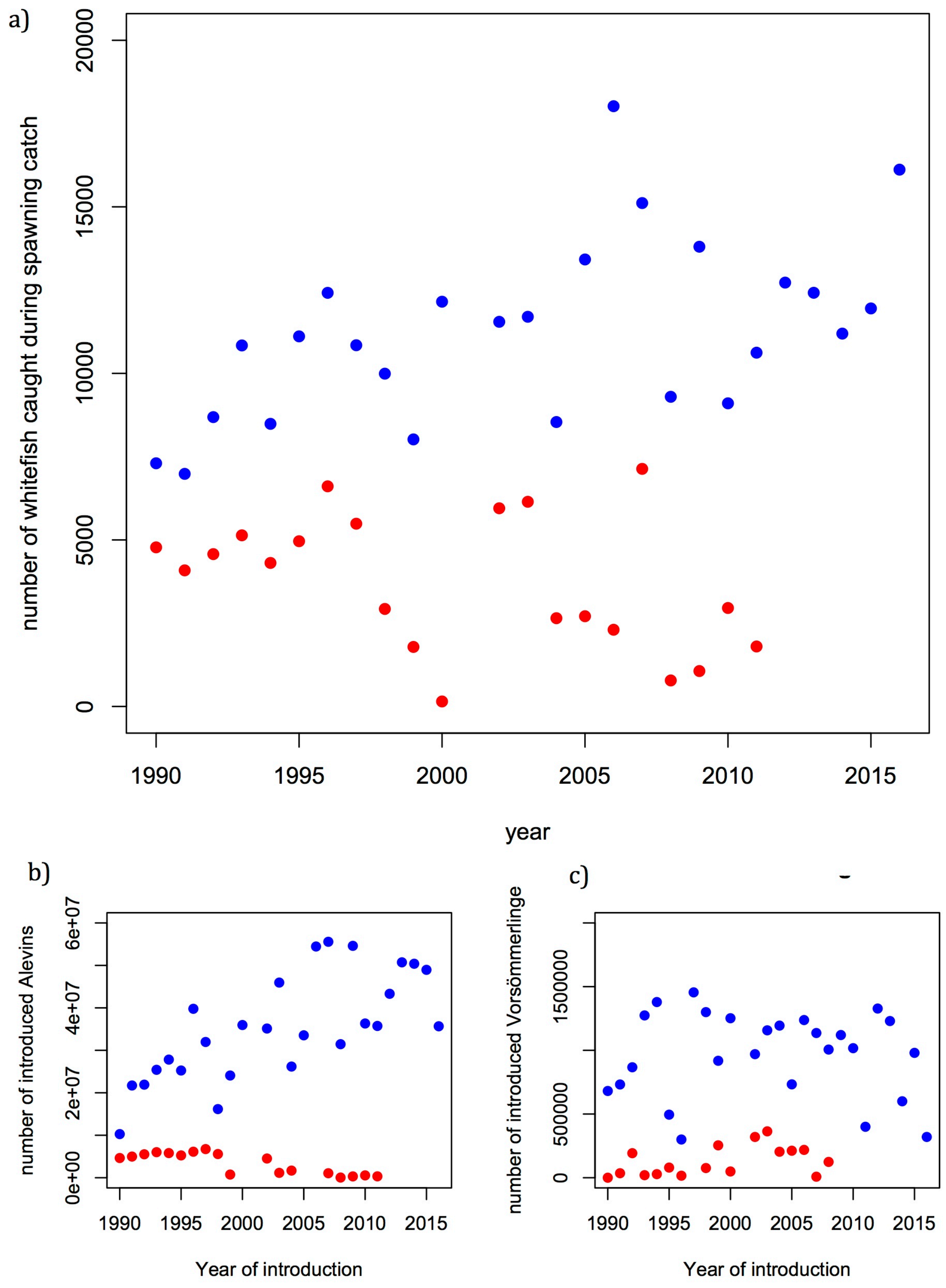

Figure S15. a) Yearly number of whitefish caught during spawning fishing in Lakes Thun (blue) and Brienz (red) from 1990 to 2016. b) Yearly number of whitefish alevins and c) whitefish fingerlings stocked into Lakes Thun (blue) and Brienz (red). Data are compiled from annual reports of the fisheries inspectorate of the Canton of Bern.

Supplementary tables

Table S1. Known whitefish species diversity in Lakes Thun (T) and Brienz (B).

Species (present name)	Common name	Lake	Native	Body size	Mean gill raker # (T/B)	spawning time	spawning depth	abundance Ω	comments
C. albellus	Brienzlig	T, B	y	small	38.1 / 41.2	summer (winter††)	deep	very abundant	
C. sp. "Felchen"	Tiefenalbock (T) Felchen (B)	T, B	y	intermediate	34.6 / 37.3	winter, before Balchen	intermediate	abundant	
C. sp. "Balchen"	Balchen	T, B	y	large	30.6 / 28.8	winter	shallow	rare	probably 2 species (this study)
C. alpinus	Kropfer	T	y	small	22.8	summer	intermediate-deep	intermediate	
C. fatioi	Albock Wanderalbock	T, B †	y	intermediate †	NA (range 34 - 39)†	september †	unknown	common on migration route	maybe extinct maybe "Felchen" or "Balchen2"
C. sp. "Albock"	Albock	T	n	intermediate	34.7	winter, before Balchen	intermediate	intermediate	introduced from Lake Constance

all data from Table S1 in Vonlanthen et al. (2012), except if indicated differently:

† Fatio (1890)

†† Local fishermen and previous reports (Steinmann, 1950; Kirchhofer, 1990) describe a winter spawning form of *C. albellus* occurring in both lakes, but its exact status remains unknown

Ω this study, Fig. S5

Table S2. Overview of the datasets included in this study.

	Year	Sampling design	Sequencer	Genotyping software	N Genetics Brienz	N Genetics Thun	N for GRN Brienz	N for GRN Thun
Bittner, PhD, 2009	2004/ 2005	Targeted	ABI 3100	Genemapper v.3.7	260	676	260	676
Vonlanthen, PhD, 2009	2004/ 2005	Targeted	CEQ 8000	Beckman Coulter	-	335	-	328
This study†	2011/ 2013	random (Projet Lac)	ABI 3130xl	Genemapper v.4.0	490	379	-	-
This study†	2014/ 15	Targeted	ABI 3130xl	Genemapper v.4.0	-	75	-	-
Vonlanthen et al., 2012 and this study††	1952-1972	Targeted	ABI 3130xl	Genemapper v.4.0	67	106	31	65
Total					817	1571	291	1069
						**2388**		**1360**

† For samples from stratified random sampling which were all genotyped in this study (N=944), total DNA was extracted from muscle or fin tissue using Chelex and proteinase K, or a QIAGEN Bio Sprint 96 extraction robot following the manufacturer's standard protocol. PCR amplification was performed using the QIAGEN Mulitplex PCR Kit in a 10 µl reaction volume of 5 µl Multiplex Master Mix, 0.5 µl Primer Mix, 3.3 µl H_2O and 1.2 µl DNA extraction product according to the following protocol: initial denaturation for 15 min at 95°C, 35 cycles of 30 sec at 94°C, 90 sec at 57°C, 90 sec at 72°C; final extension for 30 min at 72°C.

†† All scale samples from the 1950s-1970s were genotyped with the methods outlined in Vonlanthen et al. (2012).

Table S3. Genetic differentiation (F_{ST}) between clearly assigned groups (Structure assignment >0.7) within Lake Thun (below diagonal) and within Lake Brienz (above diagonal). All F_{ST}s are highly significant (p<0.001). Sample sizes are given in the first column for Lake Thun, and in the first row for Lake Brienz.

**Genetic group**	*C.* sp. "Balchen1" (31)	*C.* sp. "Balchen2" (21)	*C.* sp. "Felchen" (68)	*C. albellus* (270)	*C. alpinus*
C. sp. "Balchen 1" (61)	-	0.15	0.23	0.39	-
C. sp. "Balchen 2" (29)	0.14	-	0.12	0.31	-
C. sp. "Felchen" (74)	0.24	0.12	-	0.14	-
C. albellus (135)	0.39	0.29	0.14	-	-
C. alpinus (178)	0.26	0.24	0.23	0.29	-
C. sp. "Albock" (164)	0.18	0.18	0.2	0.28	0.12

Table S4. Locus by locus Fst for all pairwise comparisons of species from Lake Brienz. Values in bold indicate significance (p<0.05).

**Comparison**<br>**Locus**	*C. sp.* "Balchen1" - *C. sp.* "Balchen2"	*C. sp.* "Balchen1" – *C. albellus*	*C. sp.* "Balchen1" - *C. sp.* "Felchen"	*C. sp.* "Balchen2" – *C. albellus*	*C. sp.* "Balchen2" - *C. sp.* "Felchen"	*C. albellus* -*C. sp.* "Felchen"
C2-157	**0.088**	**0.108**	**0.140**	**0.023**	-0.001	**0.023**
Cocl45	0.016	**0.145**	0.002	**0.047**	-0.002	**0.079**
Cocl49	0.001	**0.088**	**0.045**	**0.052**	**0.014**	**0.031**
Cocl6	0.005	**0.417**	**0.112**	**0.411**	**0.115**	**0.147**
Cocl61	**0.059**	**0.508**	**0.434**	**0.439**	**0.371**	-0.001
Cocl68	0.023	**0.077**	**0.073**	**0.028**	**0.036**	**0.023**
BWF2	**0.158**	**0.232**	**0.181**	**0.093**	**0.028**	**0.071**
Cocl10	**0.162**	**0.505**	**0.246**	**0.486**	**0.141**	**0.154**
Cocl18	**0.379**	**0.845**	**0.630**	**0.444**	**0.128**	**0.091**
Cocl4	**0.024**	0.003	0.015	**0.020**	**0.017**	0.002

Table S5. Locus by locus Fst for all pairwise comparisons of species from Lake Thun. Values in bold indicate significance (p<0.05).

**Comparison**<br>**Locus**	*C.sp.* "Balchen1" - *C.sp.* "Balchen2"	*C.sp.* "Balchen1" -*C. albellus*	*C.sp.* "Balchen1" -*C.sp.* "Felchen"	*C.sp.* "Balchen2" -*C. albellus*	*C.sp.* "Balchen2" -*C.sp.* "Felchen"	*C. albellus* -*C.sp.* "Felchen"	*C.sp.* "Balchen1" -*C. alpinus*	*C.sp.* "Balchen2" -*C. alpinus*	*C. albellus* -*C. alinus*	*C.sp.* "Felchen"- *C. alinus*	*C.sp.* „Albock"- *C.sp.* „Balchen1"	*C.sp.* „Albock"- *C.sp.* „Balchen2"	*C.sp.* „Albock"- *C. albellus*	*C.sp.* „Albock"- *C.sp.* „Felchen"	*C.sp.* „Albock"- *C. alpinus*
C2-157	**0.008**	**0.055**	**0.059**	**0.018**	**0.027**	**0.018**	**0.064**	**0.024**	0.001	**0.014**	**0.025**	**0.007**	**0.021**	**0.044**	**0.033**
Cocl45	**0.076**	**0.206**	**0.062**	**0.034**	-0.002	**0.046**	**0.009**	**0.141**	**0.281**	**0.122**	**0.122**	**0.334**	**0.468**	**0.306**	**0.058**
Cocl49	**0.018**	**0.063**	**0.020**	**0.022**	**0.006**	**0.033**	**0.007**	**0.007**	**0.040**	**0.007**	**0.040**	**0.030**	**0.052**	**0.022**	**0.027**
Cocl6	0.004	**0.241**	**0.030**	**0.265**	**0.051**	**0.120**	0.003	**0.009**	**0.189**	**0.014**	**0.116**	**0.090**	**0.204**	**0.112**	**0.089**
Cocl61	**0.054**	**0.426**	**0.430**	**0.265**	**0.280**	0.003	**0.090**	**0.035**	**0.385**	**0.407**	**0.039**	**0.029**	**0.390**	**0.403**	**0.010**
Cocl68	**0.059**	**0.041**	**0.037**	0.001	**0.033**	**0.023**	**0.521**	**0.352**	**0.392**	**0.451**	**0.246**	**0.111**	**0.145**	**0.192**	**0.116**
BWF2	**0.077**	**0.237**	**0.204**	**0.126**	**0.068**	**0.064**	**0.198**	**0.117**	**0.049**	**0.062**	**0.192**	**0.098**	**0.135**	**0.101**	**0.118**
Cocl10	**0.084**	**0.266**	**0.126**	**0.223**	**0.043**	**0.080**	**0.465**	**0.466**	**0.088**	**0.298**	**0.153**	**0.158**	**0.029**	**0.066**	**0.124**
Cocl18	**0.274**	**0.721**	**0.522**	**0.306**	**0.099**	**0.063**	**0.288**	**0.056**	**0.410**	**0.212**	**0.135**	**0.028**	**0.401**	**0.202**	**0.077**
Cocl4	**0.037**	**0.020**	**0.024**	**0.018**	**0.013**	-0.001	**0.083**	**0.015**	**0.041**	**0.030**	**0.020**	**0.024**	**0.047**	**0.046**	**0.074**

Table S6. Proportion of unclearly assigned individuals (highest assignment proportion < 0.7) for each species and over all individuals caught during quantitative fishing in Lakes Thun and Brienz.

**Genetic group**	Thun	Brienz
C. sp. “Balchen 1”	0.800	0.143
C. sp. “Balchen 2”	0.862	0.846
C. sp. “Felchen”	0.743	0.738
C. albellus	0.594	0.435
C. alpinus	0.370	-
C. sp. “Albock”	0.563	-
overall	0.615	0.500

Table S7. P-values of Kruskal-Wallis and post-hoc dunns test for differences in spawning depth for clearly assigned groups (Structure assignment >0.7) within Lake Thun (below diagonal) and within Lake Brienz (above diagonal). P-values are adjusted for multiple testing using Holm’s method. Sample sizes are given in the first column for Lake Thun, and in the first row for Lake Brienz.

**Genetic group**	*C.* sp. “Balchen1” (28)	*C.* sp. “Balchen2” (19)	*C.* sp. “Felchen” (46)	*C. albellus* (56)	*C. alpinus*
C. sp. “Balchen 1” (49)	-	**0.005**	**0**	**0**	-
C. sp. “Balchen 2” (18)	*0.078*	-	*0.102*	**0**	-
C. sp. “Felchen” (43)	**0**	**0**	-	**0**	-
C. albellus (49)	**0**	**0**	0.303	-	-
C. alpinus (136)	**0**	**0.001**	0.104	**0.002**	-
C. sp. “Albock” (132)	*0.093*	0.210	**0**	**0**	**0**
Kruskal-Wallis test	**Thun**	X^2_5=229.0, p=0	**Brienz**	X^2_3=118.02, p=0	

Table S8. P-values of Kruskal-Wallis and post-hoc dunns test for differences in spawning time for clearly assigned groups (Structure assignment >0.7) within Lake Thun (below diagonal) and within Lake Brienz (above diagonal). P-values are adjusted for multiple testing using Holm’s method. Sample sizes are given in the first column for Lake Thun, and in the first row for Lake Brienz.

**Genetic group**	*C.* sp. “Balchen1” (28)	*C.* sp. “Balchen2” (19)	*C.* sp. “Felchen” (46)	*C. albellus* (56)	*C. alpinus*
C. sp. “Balchen 1” (49)	-	**0.005**	*0.095*	**0**	-
C. sp. “Balchen 2” (18)	*0.099*	-	**0.038**	**0**	-
C. sp. “Felchen” (43)	**0**	**0.014**	-	**0**	-
C. albellus (49)	**0**	**0**	**0.033**	-	-
C. alpinus (136)	**0**	**0**	*0.077*	0.183	-
C. sp. “Albock” (132)	0.297	0.210	**0**	**0**	**0**
Kruskal-Wallis test	**Thun**	X^2_5=217.9, p=0	**Brienz**	X^2_3=82.08, p=0	

Table S9. P-values of Kruskal-Wallis and post-hoc dunns test for differences in GRN for clearly assigned groups (Structure assignment >0.7) within Lake Thun (below diagonal) and within Lake Brienz (above diagonal). P-values are adjusted for multiple testing using Holm's method. Sample sizes are given in the first column for Lake Thun, and in the first row for Lake Brienz.

**Genetic group**	*C.* sp. "Balchen1" (28)	*C.* sp. "Balchen2" (19)	*C.* sp. "Felchen" (46)	*C. albellus* (56)	*C. alpinus*
C. sp. "Balchen 1" (60)	-	**0**	**0**	**0**	-
C. sp. "Balchen 2" (25)	**0.023**	-	*0.075*	**0**	-
C. sp. "Felchen"	**0**	**0.01**	-	**0.001**	-
C. albellus (50)	**0**	**0**	0.163	-	-
C. alpinus (136)	**0**	**0**	**0**	**0**	-
C. sp. "Albock" (146)	**0**	0.188	**0.021**	**0**	**0**
Kruskal-Wallis test	**Thun**	X^2_5=318.3, p=0	**Brienz**	X^2_3=88.2, p=0	

Table S10. P-values of Kruskal-Wallis and post-hoc dunns test for differences in habitat depth in autumn in the benthic zone for clearly assigned groups (Structure assignment >0.7) within Lake Thun (below diagonal) and within Lake Brienz (above diagonal). P-values are adjusted for multiple testing using Holm's method. Sample sizes are given in the first column for Lake Thun, and in the first row for Lake Brienz. Tests for differentiation in depth occupation in autumn could not be performed for *C.* sp. "Balchen1" and *C.* sp. "Balchen2", because of very small sample sizes in each lake.

**Genetic group**	*C.* sp. "Balchen1" (4)	*C.* sp. "Balchen2" (1)	*C.* sp. "Felchen" (12)	*C. albellus* (82)	*C. alpinus*
C. sp. "Balchen 1" (1)	-	-	-	-	-
C. sp. "Balchen 2" (2)	-	-	-	-	-
C. sp. "Felchen" (7)	-	-	-	*0.074*	-
C. albellus (28)	-	-	0.640	-	-
C. alpinus (24)	-	-	0.617	0.332	-
C. sp. "Albock" (1)	-	-	-	-	-
Kruskal-Wallis test	**Thun**	X^2_2=0.672, p=0.71	**Brienz**	W=649.5, p=0.074	

Table S11. P-values of Kruskal-Wallis and post-hoc dunns test for differences in habitat depth in autumn in the pelagic zone for clearly assigned groups (Structure assignment >0.7) within Lake Thun (below diagonal) and within Lake Brienz (above diagonal). P-values are adjusted for multiple testing using Holm's method. Sample sizes are given in the first column for Lake Thun, and in the first row for Lake Brienz. Tests for differentiation in depth occupation in autumn could not be performed for *C.* sp. "Balchen1" and *C.* sp. "Balchen2", because of very small sample sizes in each lake.

**Genetic group**	*C.* sp. "Balchen1" (2)	*C.* sp. "Balchen2" (1)	*C.* sp. "Felchen" (10)	*C. albellus* (131)	*C. alpinus*
C. sp. "Balchen 1" (0)	-	-	-	-	-
C. sp. "Balchen 2" (2)	-	-	-	-	-
C. sp. "Felchen" (11)	-	-	-	0.707	-
C. albellus (52)	-	-	0.216	-	-
C. alpinus (5)	-	-	-	-	-
C. sp. "Albock" (13)	-	-	0.296	*0.063*	-
Kruskal-Wallis test	**Thun**	X^2_2=4.9, p=0.09	**Brienz**	W=701.5, p=0.707	

Table S12. Mantel and partial Mantel correlations between genetic cluster membership and geographic distance, while correcting for spawning depth or/and spawning time. We only used contemporary individuals. For Lake Thun, we either considered individuals from all species or only those whose sum of assignment likelihoods for the four species also found in Lake Brienz was >0.85

Mantel or Partial mantel test between	Thun all	Thun 4 species	Brienz
cluster membership ~ geographic distance	0.07 ***	0.04 ***	0.12 ***
cluster membership ~ geographic distance + spawning depth	0.04 ***	0.01 ns	0 ns
cluster membership ~ geographic distance + spawning time	0.06 ***	0.02 **	0.12***
Residuals(cluster membership ~ spawning depth)~ geographic distance + spawning time	0.03 ***	-	-

Table S13. Intraspecific Mantel correlations between ecological and genetic data. Pearson correlation (r) and P-values obtained from 10000 permutations are reported. Only clearly assigned individuals were used for comparisons (maximum assignment proportion >0.7). For “Balchen1” and “Balchen2”, sample sizes of the fishing outside of spawning season are too low to compare habitat depth occupation.

species	GRN	spawning depth	spawning time	habitat depth benthic	habitat depth pelagic	Geo. Distance spawning
Thun						
C. sp. “Balchen1”	r=-0.03,p=0.67	r=0,p=0.47	r=-0.01,p=0.57	-	-	r=0.04,p=0.24
C. sp. “Balchen2”	r=0.08,p=0.21	r=0.12,p=0.11	r=-0.17,p=0.98	-	-	r=-0.13,p=0.96
C. sp. “Felchen”	0,p=0.51	**r=0.12, p=0.05**	r=0.05,p=0.12	r=-0.26,p=0.85	r=0,p=0.5	**r=0.10,p=0.03**
C. albellus	r=0.11,p=0.08	r=-0.02,p=0.57	r=0-01,p=0.45	r=-0.04,p=0.65	r=0.04,p=0.29	**r=0.17,p=0.004**
C. alpinus	r=0.05,p=0.10	r=0,p=0.56	r=0,p=0.47	r=-0.07,p=0.84	r=0.31,p=0.18	r=0.01,p=0.37
C. sp. “Albock”	r=0.01,p=0.41	r=-0.08,p=0.96	r=-0.03,p=0.71	-	r=-0.09,p=0.75	r=-0.03,p=0.84
Brienz						
C. sp. “Balchen1”	r=-0.14,p=0.94	r=-0.05,p=0.60	r=0,p=0.5	-	-	r=0.01,p=0.4
C. sp. “Balchen2”	r=0.15,p=0.14	r=0.16,p=0.08	r=0.08,p=0.19	-	-	**r=0.16,p=0.05**
C. sp. “Felchen”	r=-0.11,p=0.93	r=-0.10,p=0.96	r=-0.11,p=0.95	r=-0.11,p=0.71	r=-0.03,p=0.53	r=-0.06,p=0.86
C. albellus	r=-0.04,p=0.72	r=0.05,p=0.15	r=-0.09,p=0.94	r=0.03,p=0.22	r=0.01,p=0.43	r=-0.07,p=1

Table S14. Between lake comparisons within species. P-values obtained from Arlequin (F_{ST}) or from Wilcoxon's signed rank tests are reported (adjusted for mutliple testing). Only clearly assigned individuals were used for comparisons (maximum assignment proportion >0.7). For “Balchen1” and “Balchen2”, sample sizes of the fishing outside of spawning season are too low to compare habitat depth occupation.

species	F_{ST}	GRN	spawning depth	spawning time	habitat depth benthic	habitat depth pelagic
C. sp. “Balchen1”	0.076	0.0007	0.609	<0.0001	-	-
C. sp. “Balchen2”	0.405	0.707	1	0.707	-	-
C. sp. “Felchen”	0.405	0.81	< 0.0001	0.008	0.576	0.093
C. albellus	0.589	0.81	<0.0001	1	0.234	<0.0001

Table S15. Multilocus pairwise F_{ST} (8 markers) between *C.* sp. “Albock” from Lake Thun and the four whitefish species from Lake Constance. Data are from the pre-eutrophication period for species from Lake Constance. All F_{ST}s are highly significant (p<0.001).

**species**	*C. wartmanni*	*C. macrophthalmus*	*C. gutturosus*	*C. arenicolus*
C. sp. “Albock”	0.110	0.028	0.238	0.106

Table S16. P_{ST} and F_{ST} values calculated from spawning and ecological variables and neutral genetic markers in Lakes Thun and Brienz. 95% confidence intervals are indicated for P_{ST} values in brackets. Results of paired t-tests between F_{ST} and P_{ST} are indicated at the bottom.

species 1	species 2	F_{ST}	P_{ST} spawning depth	P_{ST} spawning time	P_{ST} GRN	P_{ST} habitat depth
**Thun**						
C. sp. “Balchen2”	*C.* sp. “Balchen1”	0.08	0.121 (0.054-0.211)	0.075(0.03-0.13)	0.183 (0.10-0.28)	0.105 (0.010-0.237)
C. sp. “Albock”*	*C.* sp. “Balchen1”	0.12	0.038 (0.005-0.104)	0.003 (0-0.02)	0.260 (0.185-0.343)	0 (0-0.319)
C. albellus	*C.* sp. “Balchen1”	0.27	0.687 (0.603-0.773)	0.470 (0.41-0.54)	0.536 (0.405-0.671)	0.061 (0.017-0.11)
C. sp. “Felchen”	*C.* sp. “Balchen1”	0.19	0.535 (0.415-0.648)	0.249 (0.18-0.33)	0.473 (0.368-0.588)	0.116 (0.056-0.191)
C. alpinus	*C.* sp. “Balchen1”	0.2	0.309 (0.239-0.376)	0.467 (0.40-0.55)	0.318 (0.232-0.426)	0.289 (0.186-0.416)
C. sp. “Albock”*	*C.* sp. “Balchen2”	0.09	0.067 (0.013-0.146)	0.047 (0.01-0.11)	0.000 (0-0.025)	0.243 (0.120-0.403)
C. albellus	*C.* sp. “Balchen2”	0.14	0.383 (0.273-0.492)	0.280 (0.21-0.36)	0.177 (0.092-0.290)	0 (0-0.051)
C. sp. “Felchen”	*C.* sp. “Balchen2”	0.07	0.215 (0.118-0.331)	0.080 (0.03-0.15)	0.097 (0.039-0.288)	0 (0-0.066)
C. alpinus	*C.* sp. “Balchen2”	0.13	0.061 (0.016-0.125)	0.265 (0.19-0.35)	0.464 (0.380-0.555)	0.089 (0-0.250)
C. albellus	*C.* sp. “Albock”*	0.21	0.675 (0.60-0.74)	0.472 (0.40-0.54)	0.178 (0.099-0.276)	0.116 (0.09-0.143)
C. sp. “Felchen”	*C.* sp. “Albock”*	0.16	0.527 (0.428-0.633)	0.241 (0.16-0.33)	0.092 (0.04-0.176)	0.200 (0.143-0.265)
C. alpinus	*C.* sp. “Albock”*	0.08	0.260 (0.20-0.32)	0.455 (0.38-0.54)	0.554 (0.471-0.637)	0.418 (0.315-0.542)
C. sp. “Felchen”	*C. albellus*	0.05	0.031 (0-0.096)	0.071 (0.02-0.14)	0.016 (0-0.070)	0.016 (0-0.074)
C. alpinus	*C. albellus*	0.21	0.178 (0.11-0.26)	0.002 (0-0.02)	0.637 (0.553-0.712)	0.131 (0.042-0.254)
C. alpinus	*C.* sp. “Felchen”	0.18	0.063 (0.016-0.136)	0.051 (0.01-0.11)	0.584 (0.500-0.669)	0.039 (0-0.150)
**Brienz**						
C. sp. “Balchen2”	*C.* sp. “Balchen1”	0.11	0.384 (0.22-0.58)	0.310 (0.17-0.47)	0.385 (0.23-0.56)	0.016 (0-0.213)
C. albellus	*C.* sp. “Balchen1”	0.34	0.951 (0.92-0.98)	0.593 (0.55-0.65)	0.820 (0.76-0.87)	0.012 (0.001-0.024)
C. sp. “Felchen”	*C.* sp. “Balchen1”	0.22	0.554 (0.45-0.67)	0.09 (0.05-0.14)	0.594 (0.47-0.71)	0.018 (0-0.045)
C. albellus	*C.* sp. “Balchen2”	0.21	0.783 (0.74-0.82)	0.625 (0.58-0.67)	0.424 (0.31-0.54)	0 (0-0.074)
C. sp. “Felchen”	*C.* sp. “Balchen2”	0.09	0.156 (0.06-0.27)	0.123 (0.08-0.18)	0.102 (0.02-0.22)	0 (0-0.047)
C. sp. “Felchen”	*C. albellus*	0.06	0.418 (0.33-0.53)	0.230 (0.12-0.37)	0.145 (0.07-0.24)	0 (0-0.027)

Table S17. Correlation coefficients for Manteltests between individual genetic distance and spawning depth (SD), geographic distance of spawning locations (Geo), spawning time (ST) or gill raker numbers (GRN), and partial Manteltests correcting for one of these factors. We only used contemporary individuals and for Lake Thun, only those whose sum of assignment likelihoods for the four species also found in Lake Brienz was >0.85. P-values are adjusted for multiple testing.

Factor used in Mantel test	SD	Geo	ST	GRN	Geo, corr. SD	ST, corr. SD	GRN, corr. SD	SD, corr. Geo	ST, corr. Geo	GRN, corr. Geo	SD, corr. ST	Geo, corr. ST	GRN, corr. ST	SD, corr. GRN	Geo, corr. GRN	ST, corr. GRN
Brienz	0.510 ***	0.124 ***	0.191 ***	0.367 ***	-0.009 ns	-0.032 ns	0.039 ns	0.498 ***	0.190 ***	0.353 ***	0.482 ***	0.122 ***	0.347 ***	0.382 ***	0.062 *	0.143 ***
Thun all	0.185 ***	0.053 ***	0.199 ***	0.127 ***	0.031 **	0.144 ***	0.113 ***	0.180 ***	0.197 ***	0.133 ***	0.124 ***	0.048 ***	0.091 ***	0.176 ***	0.065 ***	0.179 ***
Thun 4 species	0.372 ***	0.072 ***	0.385 ***	0.302 ***	0.033 ns	0.261 ***	0.176 ***	0.367 ***	0.383 ***	0.302 ***	0.239 ***	0.052 *	0.209 ***	0.285 ***	0.074 *	0.322 ***

Table S18. Correlation coefficients for partial Manteltests between individual genetic distance and genetic cluster membership, while correcting for spawning depth, geographic distance of spawning locations, gill raker numbers or all of these factors. We only used contemporary individuals and for Lake Thun, only those whose sum of assignment likelihoods for the four species also found in Lake Brienz was >0.85.

Partial mantel test between	Thun	Brienz
Genetic distance ~cluster membership + spawning depth	0.28 ***	0.20 ***
Genetic distance ~cluster membership + geography	0.35 ***	0.37 ***
Genetic distance ~cluster membership + gill raker numbers	0.31 ***	0.31 ***
Genetic distance ~cluster membership + spawning time	0.27***	0.36***
Residuals(genetics~spawning depth) ~cluster membership + geography	0.27 ***	0.18 ***
Residuals(genetics~spawning depth) ~cluster membership + gill raker numbers	0.24 ***	0.18 ***
Residuals(genetics~spawning depth) ~cluster membership + spawning time	0.21***	0.19***
Residuals(Residuals(genetics~spawning depth)~geography) ~cluster membership + spawning time	0.21 ***	0.19***
Residuals(Residuals(genetics~spawning depth)~geography) ~cluster membership + gill raker numbers	0.24 ***	0.18 ***
Residuals(Residuals(Residuals(genetics~spawning depth)~geography)~ gill raker numbers) ~cluster membership + spawning time	0.18***	0.19 ***

Table S19. Number of private alleles of Lake Constance whitefish species prior to eutrophication (8 markers), and their representation in Lake Thun. Numbers in parentheses indicate the number of markers in which private alleles were found.

**species**	*C. wartmanni* (N=42)	*C. macrophthalmus* (N=26)	*C. gutturosus* (N=59)	*C. arenicolus* (N=48)	Total (N=175)
Species specific alleles in Lake Constance species	2 (2)	16 (5)	8 (5)	0	26
Species specific alleles from Lake Constance species found in Lake Thun, but not in Lake Brienz	1	4 (2)	1	-	6

Table S20. Genetic differentiation (F_{ST}) between genetic groups (individuals are assigned based on their maximum assignment proportion) within Lake Thun for individuals with minor assignment to *C.alpinus* or "Albock" (sum of structure assignment LH to "Balchen1", "Balchen2", "Felchen", *C. albellus* >0.85). All F_{ST}s are highly significant (p<0.001).

**Genetic group**	*C.* sp. "Balchen1"	*C.* sp. "Balchen2"	*C.* sp. "Felchen"	*C. albellus*
C. sp. "Balchen1"	-			
C. sp. "Balchen 2"	0.07	-		
C. sp. "Felchen"	0.19	0.08	-	
C. albellus	0.27	0.17	0.05	-

References

Bittner, D. 2009. Gonad deformations in whitefish (*Coregonus spp.)* from Lake Thun, Switzerland – A population genetic and transcriptomic approach. PhD Thesis, University of Bern, Bern, Switzerland.

Douglas, M.R., Brunner, P. C. & Bernatchez, L. 1999. Do assemblages of *Coregonus* (Teleostei: Salmoniformes) in the Central Alpine region of Europe represent species flocks? *Mol. Ecol.* 8: 589–603.

Douglas, M. & Brunner, P. 2002. Biodiversity of central alpine Coregonus (Salmoniformes): impact of one-hundred years of management. *Ecol. Appl.* 12: 154-172.

Fatio, V. 1885. Les corégones de la Suisse (féras diverses) classification et conditions de frai. Recueil Zool. Suisse Sér. 1, 2 : 649-665, pls. 22-23.

Fatio, V. 1890. Faune des Vertébrés de la Suisse, Vol. V, 2e partie. H. Georg Libraire-éditeur, Geneva Genf und Basel.

Hardy, O.J. & Vekemans, X. 2002. SPAGEDI: a versatile computer program to analyse spatial genetic structure at the individual or population levels. *Mol. Ecol. Notes* 4: 618–620.

Hatfield, T. & Ptolemy, J. 2001. Status of the stickleback species pair, *Gasterosteus* spp., in Paxton Lake, Texada Island, British Columbia. *Can. Field. Nat.* 115: 591-596.

Heuscher J. 1901. Thuner- und Brienzer-See. Ihre biologischen und Fischerei-Verhältnisse. Zwingli, Pfäffikon.

Holm, 1979. A simple sequentially rejective multiple test procedure. *Scand. J. Stat*. 6: 65–70.

Hudson, A.G., Lundsgaard-Hansen, B., Lucek, K., Vonlanthen, P. & Seehausen, O. 2016. Managing cryptic biodiversity: fine-scale intralacustrine speciation along a benthic gradient in Alpine whitefish (*Coregonus* spp.). *Evol. Appl.* 10:251-266.

Hudson, A. G., Vonlanthen, P. & Seehausen, O. 2010. Rapid parallel adaptive radiation from a single hybridogenic ancestral population. *Proc. R. Soc. Lond. B* 278: 58-66.

Ingram, T., Hudson, A.G., Vonlanthen, P. & Seehausen, O. 2012. Does water depth or diet divergence predict progress towards ecological speciatio in whitefish radiations? *Ecol. Ecol. Res.* 14: 487-502.

Kottelat, M. 1997. European freshwater fishes. An heuristic checklist of the freshwater fishes of Europe (exlusive of former USSR), with an introduction for non-systematists and comments on nomenclature and conservation. Biologia (Bratislava), 52 (Suppl. 5): 1-271.

Oksanen, J., Blanchet, F.G., Kindt, R., Legendre, P., Minchin, P.R., O'Hara, R.B. *et al.* 2012. vegan: Community Ecology Package. R package version 2.0-4. http://CRAN.R-

project.org/package=vegan
Pritchard, J.K., Stephens, M. & Donnelly, P. 2000. Inference of population structure using multilocus genotype data. *Genetics*, **155**, 945–959.
Revell, L. J. 2012. phytools: An R package for phylogenetic comparative biology (and other things). Methods Ecol. Evol. 3 217-223.
Rosenberg, N.A., Mahajan, S., Ramachandran, S., Zhao, C., Pritchard, J.K. & Feldman, M.W. 2005. Clines, clusters, and the effect of study design on the inference of human population structure. *Public Library of Science Genetics*, **1**, 660–671.
Rousset, F. 2000. Genetic differentiation between individuals. *J. Evol. Biol.* 13: 58–62.
Serre D & Päbö S. 2004 Evidence for gradients of human genetic diversity within and among continents. Genome Res 14: 1679–1685.
Seehausen, O., Terai, Y., Magalhaes, I.S., Carleton, K.C., Mrosso, H.D.J., Miyagi, R. *et al.* 2008. Speciation through sensory drive in cichlid fish. *Nature* 455: 620–626.
Steinmann, P. 1950. Monographie der schweizerischen Koregonen. Beitrag zum Problem der Entstehung neuer Arten. Spezieller Teil. *Schweiz. Z. Hydrol.* 12: 340-491.
Vonlanthen, P., Roy, D., Hudson, A.G., Largiadèr, C. R., Bittner, D. & Seehausen, O. 2009. Divergence along a steep ecological gradient in lake whitefish (*Coregonus* sp.). *J. Evol. Biol.* 22: 498-514.
Vonlanthen, P., Bittner, D., Hudson, A., Young, K.A., Müller, R., Lundsgaard-Hansen, B. *et al.* 2012. Eutrophication causes speciation reversal in whitefish adaptive radiations. *Nature* 482: 357-363.
Vonlanthen, P. 2009. On speciation and its reversal in adaptive radiations The central European whitefish system. PhD Thesis, University of Bern, Bern, Switzerland.
Wagner, C.E., Harmon, L. & Seehausen, O. 2014. Cichlid species-area relationships are shaped by adaptive radiations that scale with area. *Ecol. Lett.* 17: 583-592.
Young, K.A., Whitman, J.M. & Turner, G.F. 2009. Secondary contact during adaptive radiation: a community matrix for Lake Malawi cichlids. *J. Evol. Biol.* 22: 882-889.
